# A Klotho-derived peptide protects against kidney fibrosis by targeting TGF-β signaling

**DOI:** 10.1038/s41467-022-28096-z

**Published:** 2022-01-21

**Authors:** Qian Yuan, Qian Ren, Li Li, Huishi Tan, Meizhi Lu, Yuan Tian, Lu Huang, Boxin Zhao, Haiyan Fu, Fan Fan Hou, Lili Zhou, Youhua Liu

**Affiliations:** 1 State Key Laboratory of Organ Failure Research, National Clinical Research Center of Kidney Disease, Division of Nephrology, Nanfang Hospital, Southern Medical University, Guangzhou, China; 2 Analysis and Test Center, Guangdong University of Technology, Guangzhou, China; 3 Department of Pharmacy, Nanfang Hospital, Southern Medical University, Guangzhou, China; 4 Bioland Laboratory (Guangzhou Regenerative Medicine and Health Guangdong Laboratory), Guangzhou, China; 5 Department of Pathology, University of Pittsburgh School of Medicine, Pittsburgh, PA USA

**Keywords:** Peptide delivery, Drug discovery, Kidney diseases

## Abstract

Loss of Klotho, an anti-aging protein, plays a critical role in the pathogenesis of chronic kidney diseases. As Klotho is a large transmembrane protein, it is challenging to harness it as a therapeutic remedy. Here we report the discovery of a Klotho-derived peptide 1 (KP1) protecting kidneys by targeting TGF-β signaling. By screening a series of peptides derived from human Klotho protein, we identified KP1 that repressed fibroblast activation by binding to TGF-β receptor 2 (TβR2) and disrupting the TGF-β/TβR2 engagement. As such, KP1 blocked TGF-β-induced activation of Smad2/3 and mitogen-activated protein kinases. In mouse models of renal fibrosis, intravenous injection of KP1 resulted in its preferential accumulation in injured kidneys. KP1 preserved kidney function, repressed TGF-β signaling, ameliorated renal fibrosis and restored endogenous Klotho expression. Together, our findings suggest that KP1 recapitulates the anti-fibrotic action of Klotho and offers a potential remedy in the fight against fibrotic kidney diseases.

## Abstract (web summary)

Klotho is an anti-ageing protein whose expression is downregulated in chronic kidney disease, but the large size of the protein makes it challenging to deliver therapeutically. Here, the authors develop a Klotho-derived peptide, and show that it recapitulates the anti-fibrotic action of Klotho and prevents kidney fibrosis in mice by targeting TGF-β signalling.

## Introduction

Chronic kidney disease (CKD) is a common and devastating disorder characterized by progressive loss of kidney function and persistent renal fibrosis, leading to the destruction of kidney parenchyma and renal failure. The prevalence of CKD is high, and about 1 in 7 adults over 30 years old suffer from CKD in the United States and the total number of individuals with CKD exceeds 800 million worldwide^1,2^. Patients with CKD are vulnerable to developing other complications such as cardiovascular diseases, hypertension, and mineral and bone disorder as well^3^. Currently, there is no effective and curative treatment for CKD except dialysis or kidney transplantation^4–6^.

Kidney fibrosis is driven primarily by α-smooth muscle actin (α-SMA)-positive myofibroblasts, which produce excessive extracellular matrix (ECM) that leads to scar formation^7–10^. Studies show that after kidney injury, interstitial fibroblasts are activated and become ECM-producing myofibroblasts^11^, in which TGF-β plays an essential role^12,13^. TGF-β transduces its signaling through binding to type 2 TGF-β receptor (TβR2), and then recruits TβR1 and activates Smad2/3. In addition, TGF-β also activates several mitogen-activated protein kinases (MAPK) including extracellular-signal-regulated kinase-1 and -2 (ERK1/2), c-Jun N-terminal kinase (JNK), and p38 MAPK^12,14^. Through both Smad-dependent and -independent pathways, TGF-β controls the expression of a diverse array of fibrosis-related genes^15–17^. In this context, it is essential to constrain TGF-β signaling for developing effective strategies against kidney fibrosis.

Klotho is an anti-aging protein that is highly expressed in kidney tubular epithelia^18,19^. It is a single-pass transmembrane protein and functions as a co-receptor for fibroblast growth factor 23 (FGF23) and plays a role in phosphate hemostasis^20^. Asides from this membranous form, there is soluble Klotho (sKlotho) containing the extracellular domain in the circulation^21^. In aging or after kidney injury, expression of Klotho is severely downregulated^18,22^. Klotho deficiency not only is the result but also the driving force of CKD^23^. Overexpression or supplementation of exogenous Klotho prevents nephropathy and attenuates renal fibrosis in various models of CKD^18,24,25^. Several potential mechanisms are postulated to contribute to the anti-fibrotic effect of Klotho in CKD, such as its inhibition on TGF-β, Wnt, and FGF2 signaling^18,24,26^.

As a membrane-bound protein, it is technically challenging and expensive to produce and use Klotho as a therapeutic remedy. In addition, high serum level of Klotho causes the disturbance of calcium and phosphorus metabolism such as hypocalcemia and hypophosphatemia^27^. However, treatment with the KL1 domain is sufficient to suppress pancreatic cancer without disturbing phosphate levels^28^. These findings prompted us to propose that small Klotho-derived peptides may mimic the anti-fibrotic action of the full-length Klotho without undesirable effects.

In this work, we synthesized a series of peptides derived from human Klotho protein and screened for their ability to inhibit TGF-β1-induced fibroblast activation. We discovered KP1, a peptide with 30 amino acids in length that inhibited TGF-β signaling by blocking TGF-β/TβR2 engagement. Our results demonstrated that KP1 is quite effective in blocking TGF-β signaling, repressing fibroblast activation, and ameliorating kidney fibrosis both in vitro and in vivo.

## Results

### Identification of a Klotho-derived peptide that blocks TGF-β signaling

**Fig. 1:**
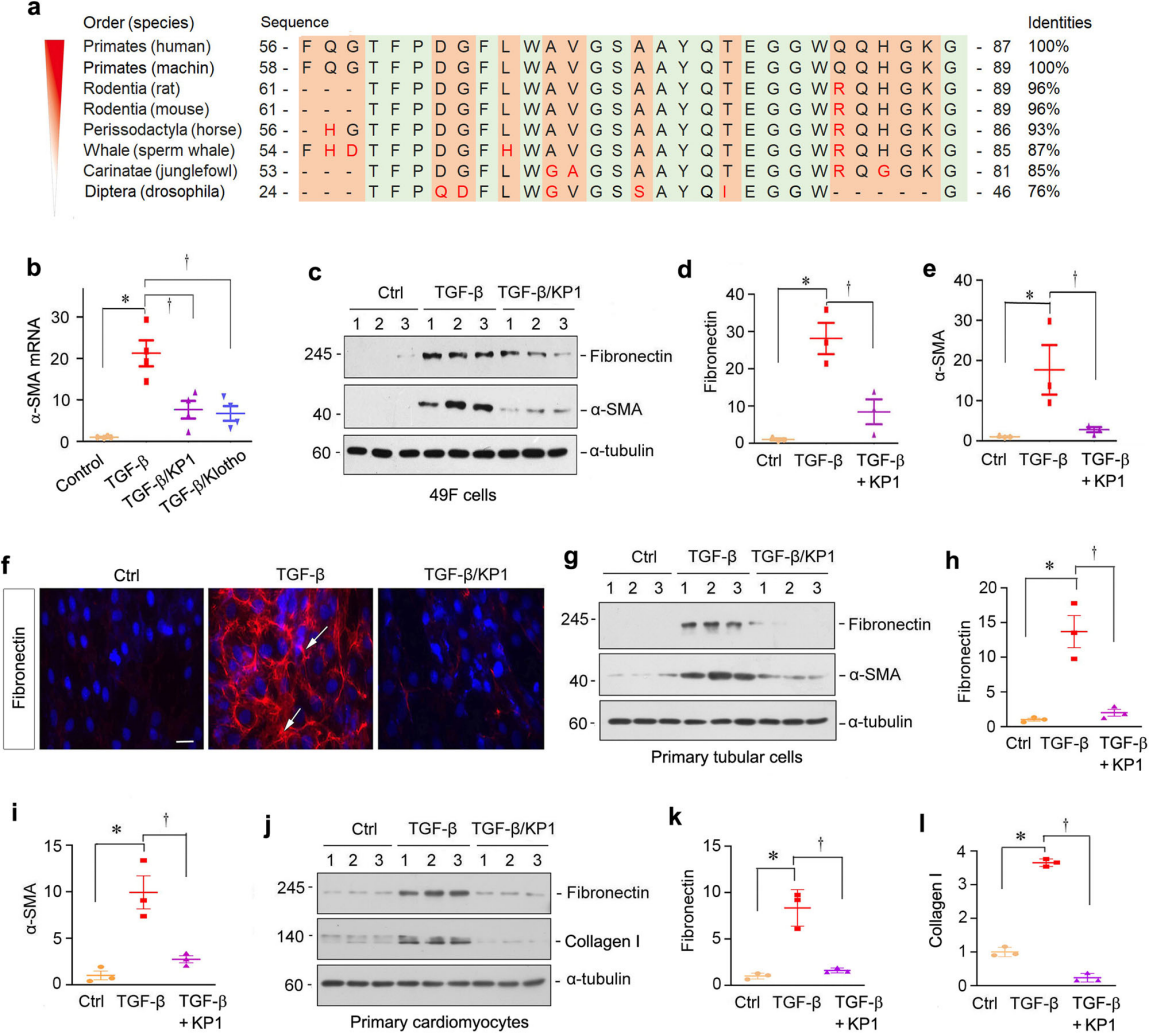
Identification of a Klotho-derived peptide (KP1) that inhibits TGF-β1 action in vitro. **a** Amino acid sequence alignment of KP1 in various species. A light green background indicates the regions with identical amino acids in different species. Differences in amino acids among various species are highlighted by red color. **b** KP1 inhibited the induction of α-SMA mRNA by TGF-β1 in NRK-49F cells. NRK-49F cells were incubated with TGF-β1 (2 ng/ml) in the absence or presence of KP1 (10 µg/ml) or sKlotho (10 µg/ml). The expression of α-SMA mRNA was assessed by qRT-PCR. *P* values (from left to right): <0.001, 0.001, <0.001. *n* = 4 biologically independent cells. **c**–**e** KP1 inhibited the protein expression of fibronectin and α-SMA. NRK-49F cells were preincubated with KP1 (10 µg/ml; 3 µM) for 1 h and then treated with TGF-β1 (2 ng/ml) for 24 h. Western blot (**c**) and quantitative data (**d**, **e**) are presented. *P* values (from left to right): 0.001 and 0.004 (fibronectin); 0.017 and 0.026 (α-SMA). *n* = 3 biologically independent cells. **f** KP1 inhibited TGF-β1-mediated fibronectin expression and deposition by NRK-49F cells. Representative micrographs with immunofluorescence staining of fibronectin are shown. Scale bar, 50 µm. Arrows indicated positive staining. **g**–**i** KP1 inhibited the expression of fibronectin and α-SMA in primary tubular cells. Western blot analyses (**g**) and quantitative data of fibronectin (**h**) and α-SMA (**i**) are shown. *P* values (from left to right): 0.001 and 0.001 (fibronectin); 0.001 and 0.003 (α-SMA). *n* = 3 biologically independent cells. **j**–**l** The protein level of fibronectin and collagen I expressed by primary cardiomyocytes. Western blot analyses (**j**) and quantitative data of fibronectin (**k**) and collagen I (**l**) are shown. *P* values (from left to right):0.001 and 0.001 (fibronectin); <0.001 and <0.001 (collagen I). *n* = 3 biologically independent cells. Ctrl controls. Data are presented as mean values ± SEM. Statistical significance was determined by one-way ANOVA followed by Fisher’s Least-significant Difference (LSD) post hoc test. Source data are provided as a Source Data file.

We first designed and synthesized 18 overlapping peptides that encompassed the KL1 domain of human Klotho protein. Using the TGF-β1-treated normal rat kidney interstitial fibroblast (NRK-49F) cells as an in vitro system, we screened these peptides for their ability to inhibit myofibroblastic activation of NRK-49F cells (Supplementary Fig. 1). After several rounds of screening, we identified a peptide with 30 amino acids encompassing from Phe57 to Gly86 of Klotho protein (Fig. 1a), designated as the Klotho-derived peptide 1 (KP1), which abolished TGF-β1 action in kidney cells. Bioinformatics analyses revealed a high degree of homology in the amino acid sequence of KP1 among different species ranging from human to fruit fly (Fig. 1a), suggesting high evolutionary conservation of KP1.

We then incubated NRK-49F cells with TGF-beta1 in the absence or presence of KP1. As shown in Fig. 1b, KP1 repressed the TGF-β1-induced mRNA expression of α-SMA, suggesting its ability to abolish the myofibroblastic activation of renal fibroblasts. The inhibitory effect of KP1 on α-SMA was comparable to soluble Klotho (sKlotho) (Fig. 1b). Similarly, KP1 inhibited α-SMA protein expression in NRK-49F cells after incubation with TGF-β1 (Fig. 1c, e). KP1 also inhibited fibronectin expression induced by TGF-β1 (Fig. 1c, d, f), as revealed by Western blotting and immunofluorescence staining.

To generalize this finding, we examined the effect of KP1 on other types of cells after TGF-β stimulation. To this end, we utilized four additional types of primary cells or cell lines from mouse, rat, and human. As shown in Fig. 1g–i and Supplementary Fig. 2a–c, KP1 also inhibited fibronectin and α-SMA expression induced by TGF-β1 in mouse primary kidney tubular epithelial cells and human proximal tubular cells (HKC-8). Similarly, KP1 abolished TGF-β1-triggered fibronectin and collagen I expression in rat primary cardiomyocytes (Fig. 1j–l) and rat primary cardiac fibroblasts (Supplementary Fig. 2d–f). Together, these results show that KP1 is capable of blocking matrix gene expression by intercepting TGF-β signaling in various types of cells.

### KP1 binds to TβR2 and disrupts TGF-β signaling

**Fig. 2:**
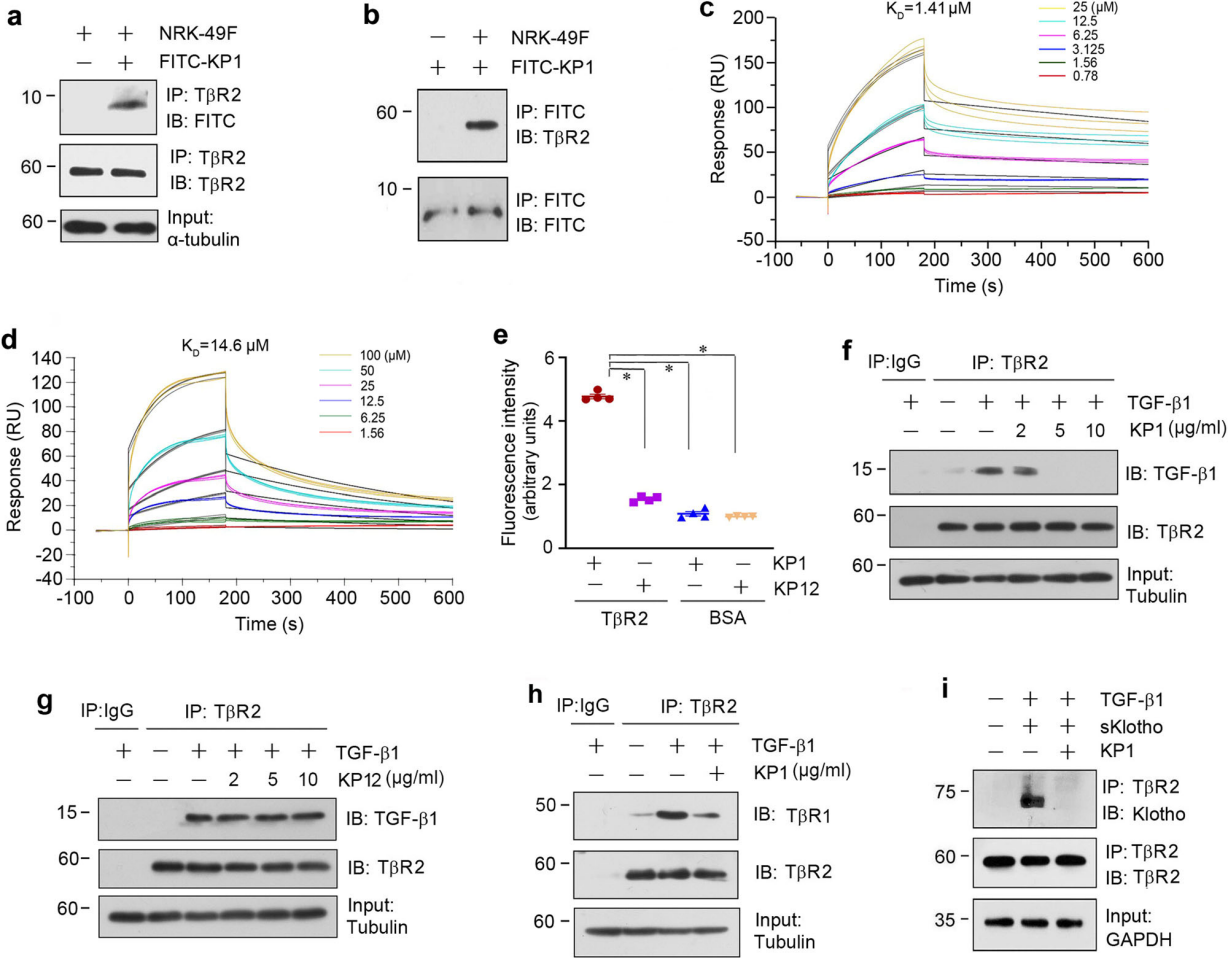
KP1 competes with TGF-β1 for TβR2 binding. **a**, **b** Co-immunoprecipitation (Co-IP) demonstrated that KP1 bound to TβR2. NRK-49F cell lysates (500 µg) and FITC-KP1 (10 µg) were immunoprecipitated (IP) with the anti-TβR2 antibody at 4 °C overnight, followed by immunoblotted (IB) for FITC and TβR2, respectively (**a**). In the reciprocal experiment, mixtures of cell lysates and FITC-KP1 were immunoprecipitated with anti-FITC, followed by immunoblotted for TβR2 and FITC (**b**). **c** The affinity of TβR2 to KP1 was shown. SPR analyses showed concentration-dependent (0.78–25 μM) binding of KP1 to TβR2 immobilized on a sensor chip. A representative sensorgram of two independent experiments is presented, in which the curves (color) with an overlay of the fitting (black) are shown. The fitted constants are *k*_a_ = 407.7 M^−1^ S^−1^; *k*_d_ = 5.8 × 10^−4^ S^−1^;  K_D_ = 1.4 × 10^−6^ M. **d** The affinity of TβR2 to KP12 was shown. SPR analyses showed concentration-dependent (1.56–100 μM) binding of KP12 to TβR2 immobilized on a sensor chip. A representative sensorgram of two independent experiments is presented, in which the curves (color) with an overlay of the fitting (black) are shown. The fitted constants are *k*_a_ = 159.5 M^−1^ S^−1^; *k*_d_ = 2.3 × 10^−3^ S^−1^; *K*_D_ = 14.6 × 10^−6^ M. **e** The specific binding of FITC-KP1 to TβR2 coated on a microplate. The fluorescence intensity of FITC-KP1 (arbitrary units) was assessed after incubating on a microplate coated with TβR2 protein. *P* values (from left to right): <0.001, <0.001, and <0.001 (one-way ANOVA with Fisher’s LSD post hoc test). (*n* = 4 biologically independent cells). Data are presented as mean values ± SEM. **f** KP1 inhibited the interaction between TβR2 and TGF-β1 dose-dependently. NRK-49F cells were pre-incubated with different amounts of KP1 for 1 h, and then treated with TGF-β1 (2 ng/ml) for 5 min. Cells were collected and immunoprecipitated with anti-TβR2 or anti-IgG. **g** Negative peptide KP12 did not block the binding of TβR2 and TGF-β1. **h** KP1 inhibited the binding of TβR1 and TβR2. **i** KP1 inhibited the interaction of TβR2 and sKlotho. Cells were transfected with sKlotho plasmid for 24 h and incubated with TGF-β1 in the absence or presence of KP1. Cells were collected and immunoprecipitated with anti-TβR2. Source data are provided as a Source Data file.

We next investigated the mechanism by which KP1 inhibits TGF-β1 signaling. As the binding of TGF-β with its type 2 receptor (TβR2) is the initial step in TGF-β signal transduction, we first examined the potential interaction between KP1 and TβR2. To this end, KP1 was labeled with fluorescein isothiocyanate (FITC) and then incubated with NRK-49F cell lysate. As shown in Fig. 2a, FITC-labeled KP1 was detected in the immunocomplexes precipitated by anti-TβR2 antibody, suggesting its interaction with TβR2. In the reciprocal experiment, TβR2 was also readily identified in the immunocomplexes precipitated by anti-FITC antibodies (Fig. 2b). To study the binding affinity of KP1 to TβR2, we employed the surface plasmon resonance (SPR) analyses with Biacore T200, an optical and label-free technique used to measure molecular interactions in real time^29–31^. As shown in Fig. 2c, the binding affinity between KP1 and TβR2 was strong and the equilibrium dissociation constant (K_D_) calculated was 1.41 µM, whereas KP12, a negative control peptide that did not inhibit TGF-β action (Supplementary Fig. 1), showed less affinity with TβR2. The K_D_ between KP12 and TβR2 was 14.6 μM (Fig. 2d, Supplementary Table 1). We also assessed the binding between KP1 and TβR2 by directly incubating FITC-KP1 on a TβR2-coated microplate. As shown in Fig. 2e, significant binding of FITC-KP1 to TβR2 was evident, as a substantial amount of fluorescence was detected after washing. Under the same conditions, much less FITC-KP12 was detected when it was incubated on the TβR2-coated microplate (Fig. 2e). As a native control, neither FITC-KP1 nor FITC-KP12 bound to bovine serum albumin (BSA) coated on the microplate (Fig. 2e), suggesting the specificity of KP1 and TβR2 interaction.

To investigate the functional consequence of KP1 and TβR2 interaction, we examined its influence on the binding of TGF-β and TβR2. To this end, NRK-49F cells were pretreated with KP1 and then incubated with TGF-β1. As shown in Fig. 2f, TGF-β1 was able to bind to TβR2 and formed a complex with it, as revealed by co-immunoprecipitation. However, such TGF-β1/ TβR2 interaction was abolished by incubation with KP1 in a dose-dependent manner (Fig. 2f), suggesting that KP1 blocks TGF-β signaling by disrupting the ligand–receptor engagement. Under the same conditions, KP12 was unable to disrupt the TGF-β1/TβR2 interaction (Fig. 2g). Consistently, KP12 failed to inhibit TGF-β1-induced fibronectin and α-SMA expression in NRK-49F cells (Supplementary Fig. 3a–c). TβR2 forms heterodimer with TβR1 after TGF-β1 binding to TβR2^12^. Thus, we tested whether KP1 inhibited the binding and recruitment of TβR1. As shown in Fig. 2h, KP1 interrupted the binding of TβR1 and TβR2. As Klotho is also able to bind to TβR2, we further examined whether KP1 can compete with it for binding. As shown in Fig. 2i, sKlotho was able to interact with TβR2, which was abolished in the presence of KP1. These results suggest that KP1 acts as an antagonist of TGF-β signaling by binding to TβR2 and disrupting TGF-β/TβR2 engagement.

### KP1 inhibits multiple downstream signaling of TGF-β in vitro

**Fig. 3:**
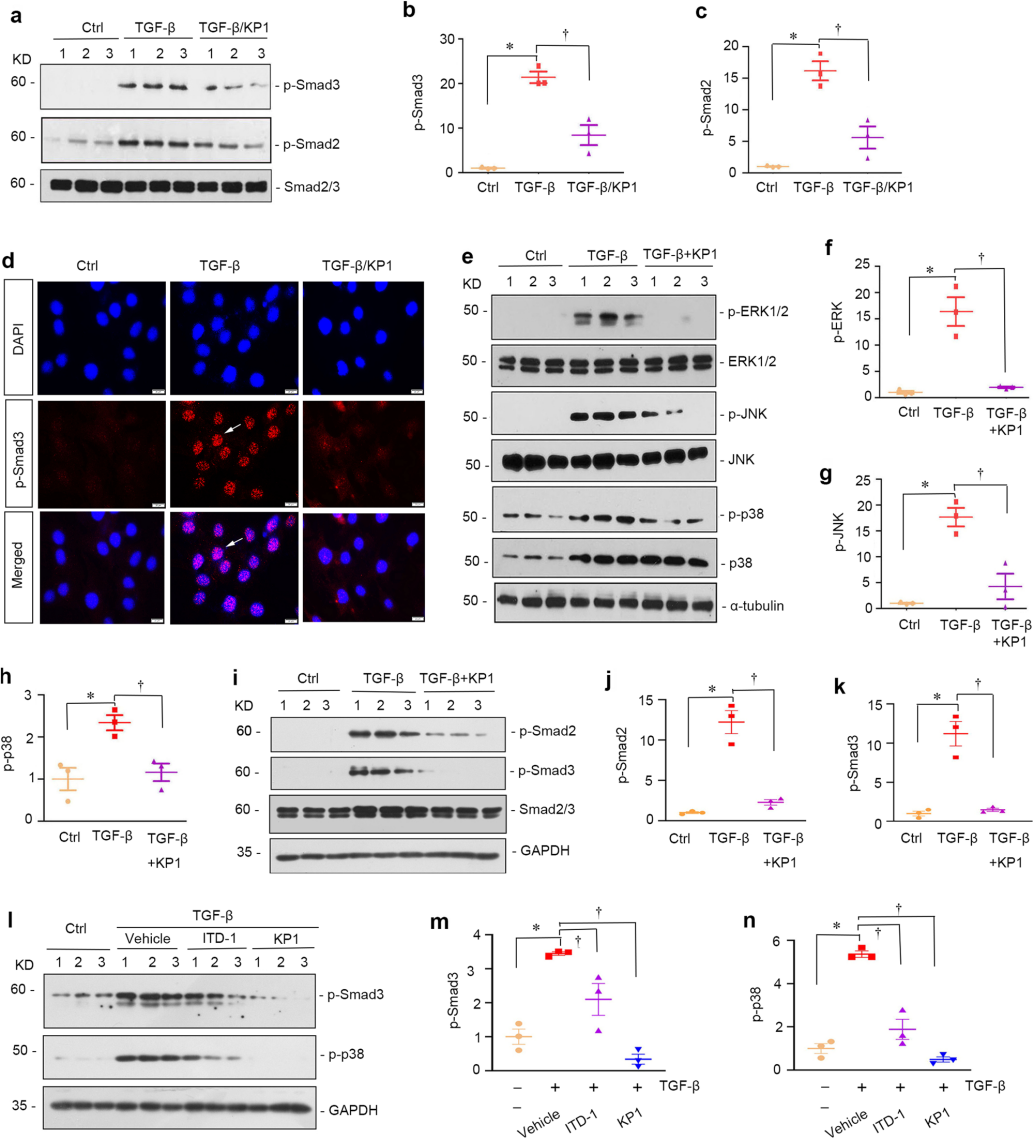
KP1 inhibits multiple signaling triggered by TGF-β1 in vitro. **a** KP1 inhibited the phosphorylation of Smad2 and Smad3 induced by TGF-β1. Numbers (1–3) indicate each individual wells of cells. Serum-starved NRK-49F cells were pre-treated with KP1 or vehicle for 1 h and then stimulated by TGF-β1 for 45 min. **b**, **c** Quantitative data of p-Smad3 (**b**) and p-Smad2 (**c**) in different groups are shown. *P* values (from left to right): <0.001 and 0.001 (p-Smad3); <0.001 and 0.001 (p-Smad2). *n* = 3 biologically independent cells. **d** Immunofluorescence staining of p-Smad3 showed that KP1 blocked TGF-β1-mediated Smad3 phosphorylation and nuclear translocation. DAPI denotes the nuclei. Arrows indicate p-Smad3 positive cells. Scale bar, 20 µm. **e**–**h** KP1 inhibited TGF-β1-induced phosphorylation of ERK1/2, JNK, and p38. Western blot analyses (**e**) and quantitative data of p-ERK1/2 (**f**), p-JNK (**g**), and p-p38 (**h**) are shown. *P* values (from left to right): <0.001 and 0.001 (p-ERK1/2); 0.001 and 0.002 (p-JNK); 0.005 and 0.010 (p-p38). *n* = 3 biologically independent cells. **i**–**k** KP1 inhibited TGF-β/Smad signaling in primary tubular cells. Western blot analyses (**i**) and quantitative data of p-Smad2 (**j**) and p-Smad3 (**k**) in different groups are shown. *P* values (from left to right): 0.032 and 0.034 (p-Smad2); 0.041 and 0.049 (p-Smad3). *n* = 3 biologically independent cells (**l**–**n**) KP1 was more effective in inhibiting TGF-β signaling compared to TβR2 inhibitor ITD-1. NRK-49F cells were pre-incubated with KP1 (10 µg/ml, 3 μM) or ITD-1 (3 μM) for 1 h, and then treated with TGF-β1 (2 ng/ml) for 45 min. Western blot analyses (**l**) and quantitative data of p-Smad3 (**m**) and p-p38 (**n**) are shown. *P* values (from left to right): *<*0.001, 0.008 and <0.001 (p-Smad3); <0.001, <0.001 and <0.001 (p-p38). *n* = 3 biologically independent cells. Ctrl controls. Data are presented as mean values ± SEM. *P* values were calculated by one-way ANOVA with Fisher’s LSD post hoc test (**b**, **c**, **f**, **g**, **h**, **m**, **n**) or Dunnett’s T3 test (**j**, **k**). Source data are provided as a Source Data file.

We further investigated the effect of KP1 on the downstream signaling of TGF-β. We first assessed the phosphorylation and activation of Smad-2 and -3 (Smad2/3), the canonical pathway mediating TGF-β actions. As shown in Fig. 3a, Smad2/3 was phosphorylated and activated after incubation with TGF-β1 in NRK-49F cells, which was inhibited by KP1. Quantitative determination of p-Smad2/3 protein levels in different groups was presented in Fig. 3b, c. Immunofluorescent staining revealed that p-Smad3 was accumulated in the nuclei of NRK-49F cells after TGF-β1 stimulation (Fig. 3d). However, incubation with KP1 largely abolished the TGF-β1-triggered p-Smad3 nuclear accumulation (Fig. 3d).

We also examined the effect of KP1 on MAPK activation induced by TGF-β. As shown in Fig. 3e–h, TGF-β1 induced the phosphorylation of ERK1/2, p38 MAPK, and JNK in NRK-49F cells, whereas KP1 hampered the phosphorylation and activation of these MAPKs (Fig. 3e–h). However, KP12 did not affect Smad2/3, ERK1/2, JNK, and p38 MAPK activation induced by TGF-β1 in NRK-49F cells (Supplementary Fig. 3d–j). KP1 also blocked TGF-β1-induced Smad2/3 phosphorylation in mouse primary tubular epithelial cells (Fig. 3i–k), rat primary cardiac fibroblasts (Supplementary Fig. 4a–c), and rat primary cardiomyocytes (Supplementary Fig. 4d–f). Furthermore, KP1 was more potent than ITD-1, a small molecule inhibitor of TGF-β signaling by inducing proteasomal degradation of the TβR2^32,33^, in blocking Smad and MAPK activation (Fig. 3l–n). Collectively, these data demonstrate that KP1 is a potent inhibitor that blocks multiple downstream signaling of TGF-β in vitro.

### KP1 preserves kidney function and reduces renal fibrosis after ischemia-reperfusion injury

**Fig. 4:**
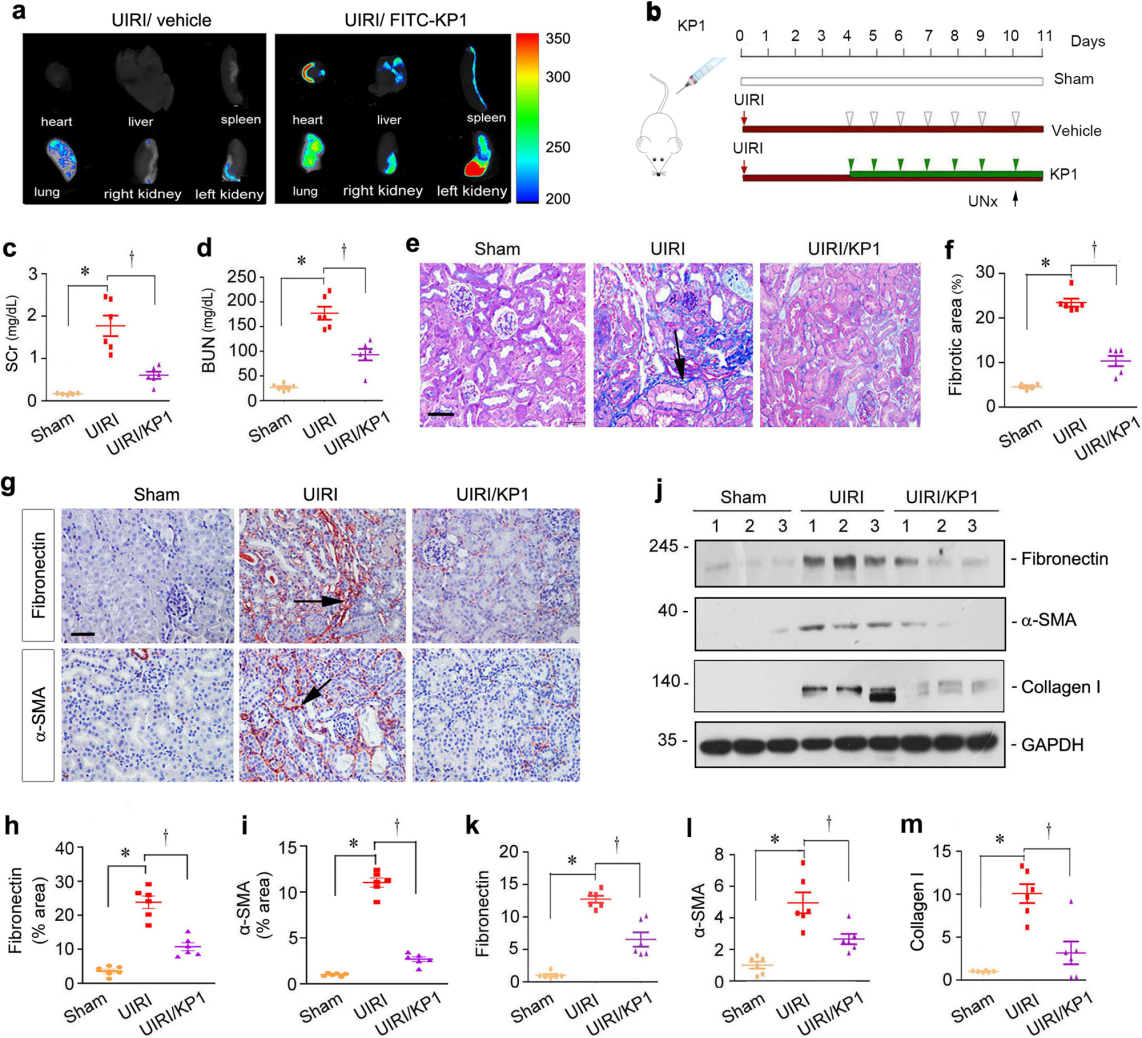
KP1 preserves renal functions and ameliorates renal fibrosis in UIRI mice. **a** Organ imaging showed that KP1 was preferentially accumulated in the injured kidney after UIRI. Relative levels of FITC-KP1 are shown in major organs. **b** Schematic diagram of the experimental design. The red line indicates the duration of surgery. The green arrowheads indicate the injections of KP1 (1 mg/kg body weight), whereas white arrowheads denote the injections of vehicle (0.01 M acetic acid). **c**, **d** KP1 preserved kidney function in UIRI mice. Serum creatinine (Scr) (**c**) and blood urea nitrogen (BUN) (**d**) levels are shown. *P* values (from left to right): 0.003, 0.010 (Scr); <0.001, *P* < 0.001 (BUN). *n* = 6 biologically independent animals. **e** Representative micrographs of Masson’s trichrome staining in different groups. Scale bar, 50 µm. Arrow indicates collagens deposition. **f** Quantitative determination of renal fibrotic lesions. *P* values (from left to right): <0.001, <0.001. *n* = 6 biologically independent animals. **g** Representative micrographs of immunohistochemical staining for fibronectin and α-SMA. Scale bar, 50 µm. **h**, **i** Quantitative data of fibronectin and α-SMA positive area in each high-power field. *P* values (from left to right): <0.001, <0.001 (fibronectin); <0.001, <0.001 (α-SMA). *n* = 6 biologically independent animals. **j** Representative Western blot showed renal protein levels of fibronectin, α-SMA, and collagen I in different groups. Numbers (1–3) indicate each individual animal in a given group. **k**–**m** Quantitative data of fibronectin (**k**), α-SMA (**l**), and collagen I (**m**) proteins in different groups. *P* values (from left to right): <0.001, 0.004 (fibronectin); 0.003, 0.045 (α-SMA); 0.001, 0.018 (collagen I). *n* = 6 biologically independent animals. ^*^P < 0.05 versus sham; ^†^*P* < 0.05 vs. UIRI alone. *P* values were determined by one-way ANOVA followed by Fisher’s LSD post hoc test (**d**, **f**, **h**, **i**) or Dunnett’s T3 test (**c**, **k**, **l**, **m**). Data are presented as mean values ± SEM. Source data are provided as a Source Data file.

In view of the effectiveness of KP1 in blocking TGF-β signaling, we sought to investigate its efficacy in ameliorating renal fibrosis in vivo. To this end, we first examined the tissue distribution of KP1 in vivo after intravenous injection. At 7 days after unilateral ischemia-reperfusion injury (UIRI), FITC-labeled KP1 was injected into UIRI mice through the tail vein. After 0.5 h, major organs of mice were collected for detecting the FITC fluorescence under in vivo imaging system. As shown in Fig. 4a, FITC-labeled KP1 was largely accumulated in the injured kidney, whereas other major organs including liver, lung, heart, and spleen, as well as the contralateral uninjured kidney, exhibited little accumulation of the FITC-labeled KP1, suggesting that KP1 is preferentially delivered to the diseased kidney.

We then employed a mouse model of UIRI and injected KP1 through daily intravenous injections, starting at 4 days after UIRI. As shown in Fig. 4b, at 10 days after UIRI, the uninjured kidney was removed through uninephrectomy (UNx) and experiments terminated at 11 days. To evaluate the therapeutic effect of KP1, we first examined renal function by measuring serum creatinine (SCr) and blood urea nitrogen (BUN). As shown in Fig. 4c, d, SCr and BUN were elevated in mice after UIRI, which were reduced after KP1 treatments. As the side effect of Klotho is hypophosphatemia and hypocalcemia^34^, blood phosphorus, and calcium level were examined. As shown in Supplementary Table 2, KP1 did not affect blood phosphorus and calcium level compared to the UIRI group. These results indicate that KP1 preserves kidney function after UIRI.

We next assessed the kidney fibrotic lesions in UIRI mice after treatment with KP1. As shown in Fig. 4e, f, Masson’s trichrome staining (MTS) revealed a substantial collagen deposition in the renal interstitial area at 11 days after UIRI, whereas KP1 alleviated the accumulation and deposition of interstitial collagens. Consistently, UIRI induced renal fibronectin accumulation and myofibroblast activation as assessed by immunohistochemical staining for fibronectin and α-SMA, both of which were mitigated by KP1 (Fig. 4g–i). Similar results were obtained when kidney lysates were analyzed for the expression of fibronectin, α-SMA, and collagen I proteins by Western blotting (Fig. 4j). Quantitative determination of fibronectin, α-SMA, and collagen I protein levels are presented in Fig. 4k–m, respectively. In short, these results indicate that KP1 ameliorates renal fibrotic lesions in a mouse model of IRI.

### KP1 inhibits TGF-β signaling in UIRI mice in vivo

**Fig. 5:**
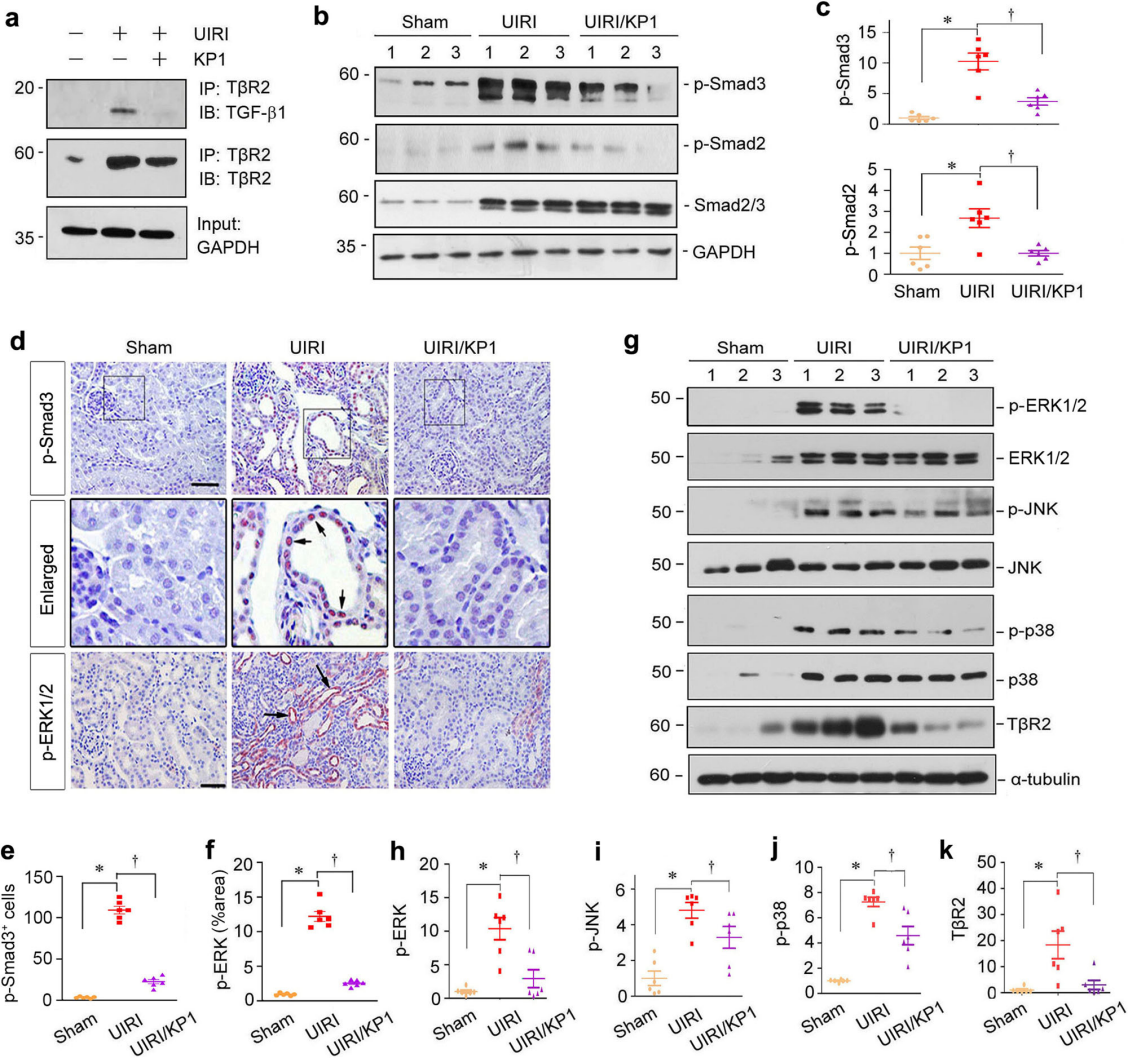
KP1 inhibits TGF-β signaling in UIRI mice. **a** Co-immunoprecipitation showed that KP1 blocked the interaction between TGF-β1 and TβR2 in vivo. **b**, **c** KP1 inhibited Smad2/3 activation in vivo. Representative Western blot analyses (**b**) and quantitative data (**c**) of p-Smad3 and p-Smad2 are shown. *P* values: <0.001, <0.001 (p-Smad3); 0.002, 0.002 (p-Smad2). *n* = 6 biologically independent animals. **d** Representative micrographs of immunohistochemical staining of p-Smad3 and p-ERK1/2. Boxed areas are enlarged. Arrows indicate positive staining. Scale bar, 50 µm. **e**, **f** Quantitative data of p-Smad3 positive cells number (**e**) and p-ERK positive staining area (**f**) in each high-power field. *P* values: *<*0.001, *<*0.001 (p-Smad3); *<*0.001, *<*0.001 (p-ERK); *n* = 6 biologically independent animals. **g** KP1 blocked the activation of MAPK and TβR2 expression in vivo. **h**–**k** Quantitative data of p-ERK1/2 (**h**), p-JNK (**i**), p-p38 (**j**), and TβR2 (**k**) are shown. *P* values (from left to right): 0.006, 0.018 (p-ERK1/2); *<*0.001, 0.046 (p-JNK); < 0.001, 0.035 (p-p38); 0.002, 0.004 (TβR2). *n* = 6 biologically independent animals. Data are presented as mean values ± SEM. Statistical significance was determined by one-way ANOVA followed by Fisher’s LSD post hoc test (**c**, **i**, **k**) or Dunnett’s T3 test (**e**, **f**, **h**, **j**). Source data are provided as a Source Data file.

We then investigated the mechanism underlying the anti-fibrotic action of KP1. Based on the in vitro data (Fig. 2), we first examined whether KP1 affects the engagement of TGF-β and TβR2 in the diseased kidney in vivo. As shown in Fig. 5a, co-immunoprecipitation revealed a substantial binding between TGF-β1 and TβR2 at 11 days after UIRI. However, such ligand-receptor interaction virtually vanished after KP1 treatment (Fig. 5a), suggesting that, similar to in vitro situation, KP1 also disrupts the engagement of TGF-β with TβR2 in vivo.

To further corroborate the inhibitory effects of KP1 on TGF-β signaling, we examined the downstream signaling of TGF-β. As shown in Fig. 5b, c, KP1 effectively inhibited Smad2/3 phosphorylation and activation in the diseased kidney, although it did not affect total Smad2/3 abundances after UIRI. Immunohistochemical staining exhibited that p-Smad3 was markedly induced and predominantly localized in the nuclei of tubular epithelial cells in the UIRI kidney, which was abolished by KP1 (Fig. 5d, e). Similarly, KP1 also repressed the phosphorylation and activation of ERK1/2, p38 MAPK, or JNK in the kidneys after UIRI (Fig. 5d, f, g–j). Of note, renal expression of TβR2 was also suppressed by KP1 in the injured kidney after UIRI (Fig. 5g, k).

### KP1 ameliorates renal fibrosis and inflammation in obstructive nephropathy

**Fig. 6:**
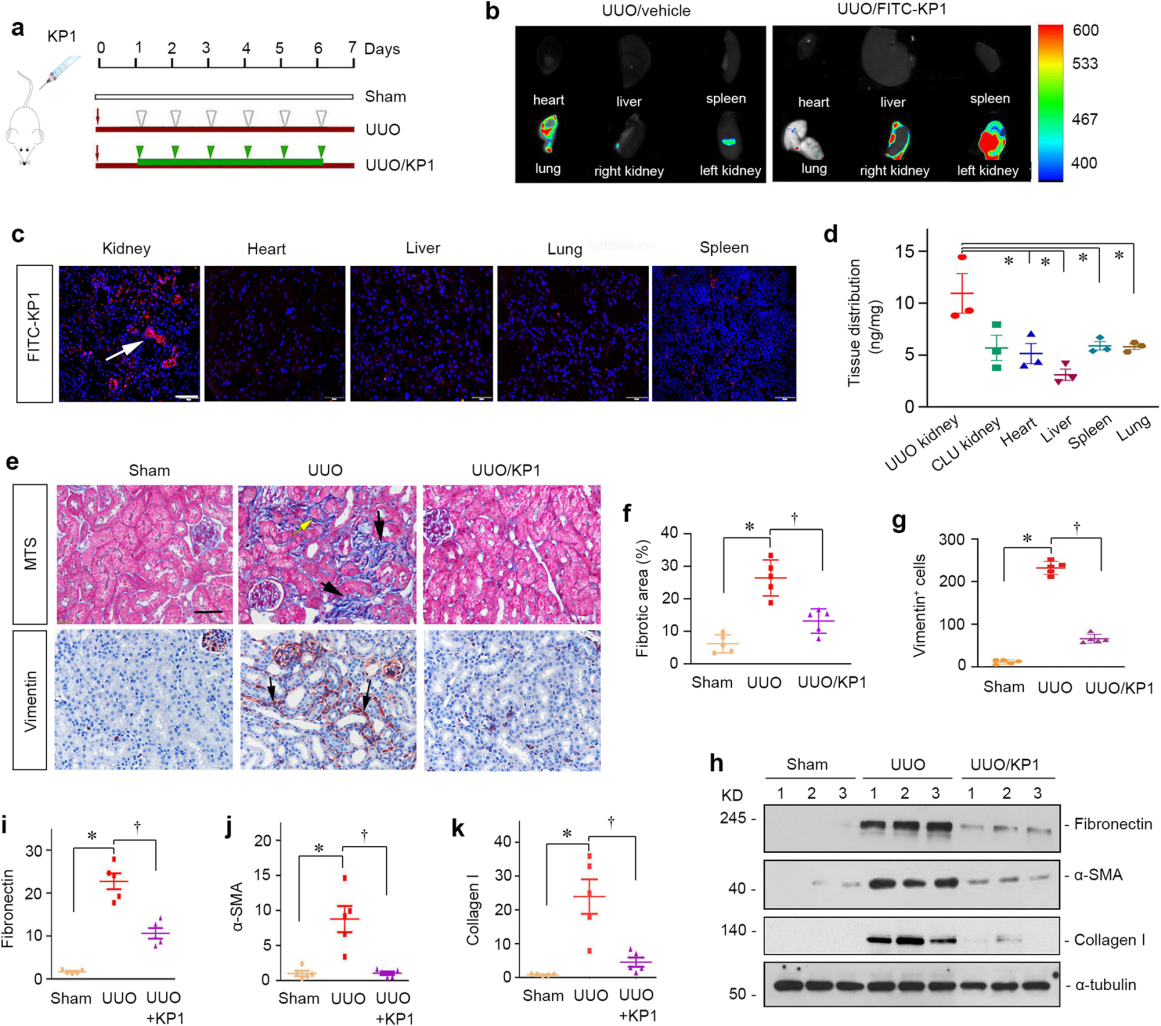
KP1 attenuates renal fibrosis in obstructive nephropathy. **a** Experimental design. The green arrowheads indicate the injections of KP1 (1 mg/kg body weight), whereas the white arrowheads indicate the injections of vehicle (0.01 M acetic acid). **b** The organ distribution of FITC-KP1 in vivo. Major organs as indicated were assessed for FITC-KP1 accumulation at 0.5 h after intravenous injection. **c** Representative micrographs of immunofluorescence of FITC-KP1 in organs. UUO mice were sacrificed 0.5 h after intravenous injection of FITC-KP1. Tissue cryosections were stained with an anti-FITC antibody. Kidney indicates UUO injured kidney. Scale bar, 50 µm. **d** The concentration of KP1 in each organ was assessed by LC–MS/MS at 0.5 h after intravenous injection. Two-sided *t* test *P* values (from left to right): 0.041, 0.014, 0.049, 0.049. *n* = 3 biologically independent animals. **e** Representative micrographs of Masson’s trichrome staining and immunohistochemical staining for vimentin showed that KP1 decreased interstitial matrix deposition and myofibroblasts activation. Arrows indicate positive staining. Scale bar, 50 µm. **f**, **g** Quantitative data of Masson’s trichrome staining and vimentin-positive cells number. *P* values: *<*0.001, *<*0.001 (Masson); *<*0.001, *<*0.001 (vimentin). *n* = 5 biologically independent animals. **h**–**k** Representative Western blot (**h**) showed renal expression of fibronectin, α-SMA, and collagen I in different groups. Quantitative data of renal fibronectin (**i**), α-SMA (**j**), and collagen I (**k**). *P* values (from left to right): 0.001, 0.001 (fibronectin); 0.033, 0.035 (α-SMA);0.026, 0.043 (collagen I). *n* = 5 biologically independent animals. Data are presented as mean values ± SEM. *P* values were determined by one-way ANOVA with Fisher’s LSD post hoc test in (**f**, **g**) or one-way ANOVA with Dunnett’s T3 test in (**i**–**k**). Source data are provided as a Source Data file.

To generalize the effect of KP1 on renal fibrosis, we sought to use another model by employing unilateral ureter obstruction (UUO), the most aggressive type of kidney fibrogenesis. As shown in Fig. 6a, mice were subjected to UUO for 7 days and KP1 was injected from day 1 to day 6. Organ distribution of FITC-labeled KP1 showed that KP1 was largely accumulated in the obstructed kidney, but not in other major organs and the contralateral unobstructed kidney (Fig. 6b). Tissue sections showed that FITC-KP1 was mainly localized in renal tubular epithelia of the obstructed kidney, but little was observed in other organs such as heart, liver, lung, and spleen (Fig. 6c). Similar results were obtained when KP1 was quantitatively assessed by liquid chromatography–tandem mass spectrometry (LC–MS/MS) analyses (Fig. 6d).

Obstructive injury-induced substantial accumulation of collagens and increased fibroblast activation in the obstructed kidney, as demonstrated by MTS and immunohistochemical staining for vimentin (Fig. 6e–g). However, KP1 ameliorated these fibrotic lesions. Analyses of protein expressions of fibronectin, α-SMA, and collagen I by Western blotting gave rise to similar results (Fig. 6h). Quantitative determination of renal fibronectin, α-SMA, and collagen I abundances is presented in Fig. 6i–k.

**Fig. 7:**
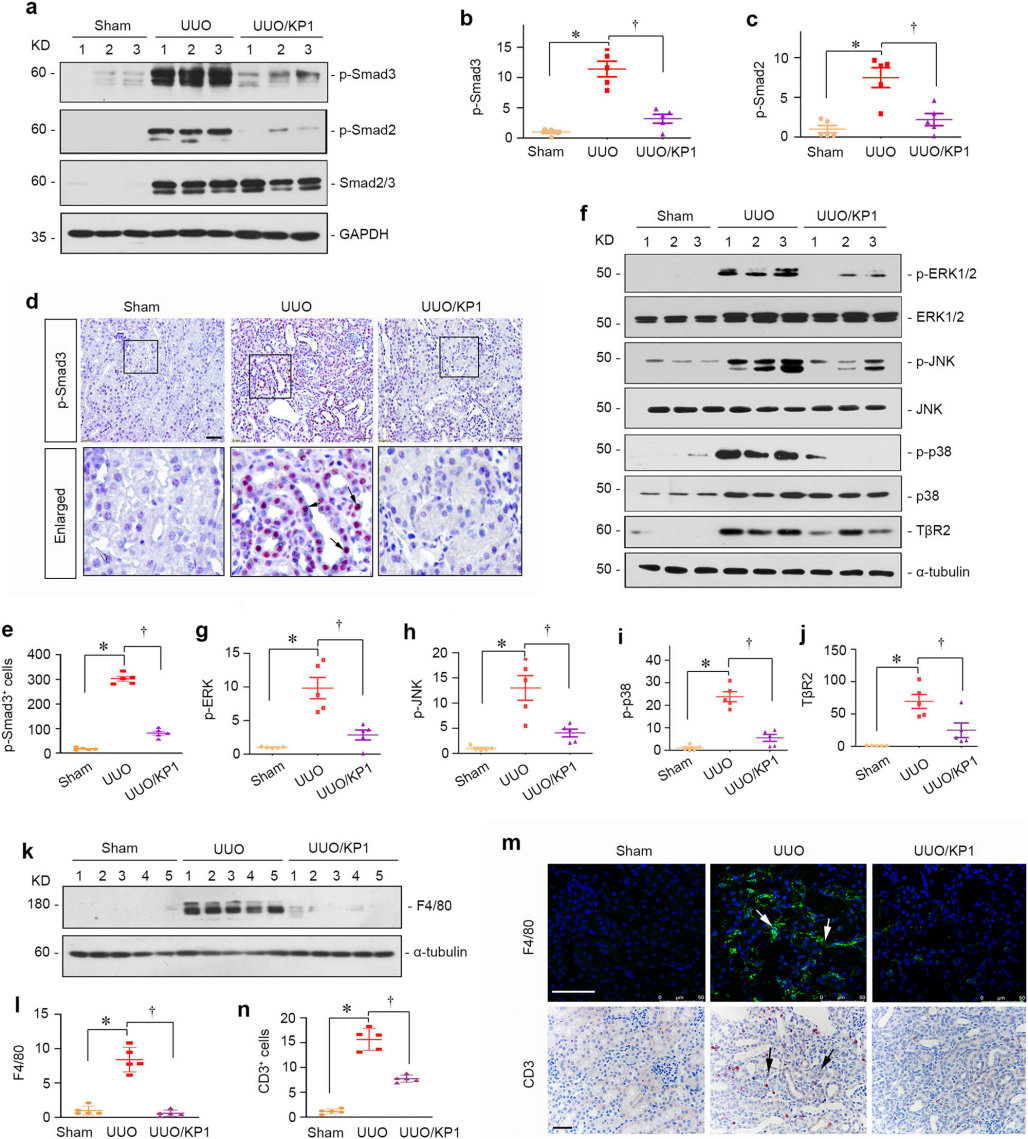
KP1 blocks TGF-β signaling and inhibits renal inflammation in UUO mice. **a**–**c** KP1 inhibited activation of Smad signaling in the UUO kidney. Representative Western blot (**a**) and quantitative data of p-Smad3 (**b**) and p-Smad2 (**c**) in the obstructed kidney are shown. *P* values (from left to right): 0.003, 0.003 (p-Smad3); *<*0.001, 0.001 (p-Smad2). *n* = 5 biologically independent animals. **d** Immunohistochemical staining of p-Smad3 in the obstructed kidney in different groups as indicated. Arrows indicate the nuclear staining of p-Smad3. Scale bar, 50 µm. **e** Quantitative data of p-Smad3 positive cells number. *P* values (from left to right): <0.001, <0.001. *n* = 5 biologically independent animals. **f** KP1 inhibited MAPK activation and TβR2 expression in UUO. **g**–**j** Quantitative data of p-ERK1/2 (**g**), p-JNK (**h**), p-p38 (**I**), and TβR2 (**j**). *P* values (from left to right): 0.013, 0.022 (p-ERK); 0.020, 0.049 (p-JNK); 0.001, 0.001 (p-p38); <0.001, 0.004 (TβR2). *n* = 5 biologically independent animals. **k**, **l** Representative Western blot (**k**) and quantitative data of F4/80 (**l**). *P* values (from left to right): 0.001, 0.001. *n* = 5 biologically independent animals. **m** Immunohistochemical staining of CD3 and immunofluorescence of F4/80 are shown. Scale bar, 50 µm. **n** Quantitative data of CD3 positive T cells number. *P* values (from left to right): <0.001, 0.002. *n* = 5 biologically independent animals. Data are presented as mean values ± SEM. *P* values were calculated by one-way ANOVA with Fisher’s LSD post hoc test (**c**, **e**, **j**) or Dunnett’s T3 test (**b**, **g**–**i**, **l**, **m**). Source data are provided as a Source Data file.

We further examined the effect of KP1 on TGF-β signaling in the UUO model. As shown in Fig. 7a–c, KP-1 did not affect Smad2/3 abundances but markedly reduced the levels of p-Smad2 and p-Smad3 in the obstructed kidney, suggesting its inhibition of TGF-β canonical signaling. Immunostaining revealed that p-Smad3 was primarily localized in the nuclei of tubular epithelial cells after UUO, which was abolished by KP1 (Fig. 7d, e). Similarly, KP1 also hampered the phosphorylation and activation of ERK1/2, p38 MAPK, and JNK in the obstructed kidney (Fig. 7f–i), and reduced TβR2 expression (Fig. 7f, j). We also compared the efficacy of KP1 and ITD-1 for their ability to inhibit TGF-β signaling and renal fibrosis in vivo. As shown in Supplementary Fig. 5, KP1 was clearly more potent than ITD-1 in blocking TGF-β signaling and alleviating fibrotic lesions after UUO. KP1 was comparable with Klotho in inhibiting TGF-β signaling and renal fibrosis after UUO (Supplementary Fig. 6).

We further examined the effect of KP1 on renal inflammation after UUO. As shown in Fig. 7k, l, UUO induced renal expression of F4/80, a marker of macrophages, and KP1 completely abolished its induction. Immunohistochemical staining also revealed that KP1 blocked renal infiltration of F4/80^+^ macrophages and CD3^+^ T cells in the obstructed kidneys (Fig. 7m, n).

### KP1 restores endogenous Klotho expression in vivo

**Fig. 8:**
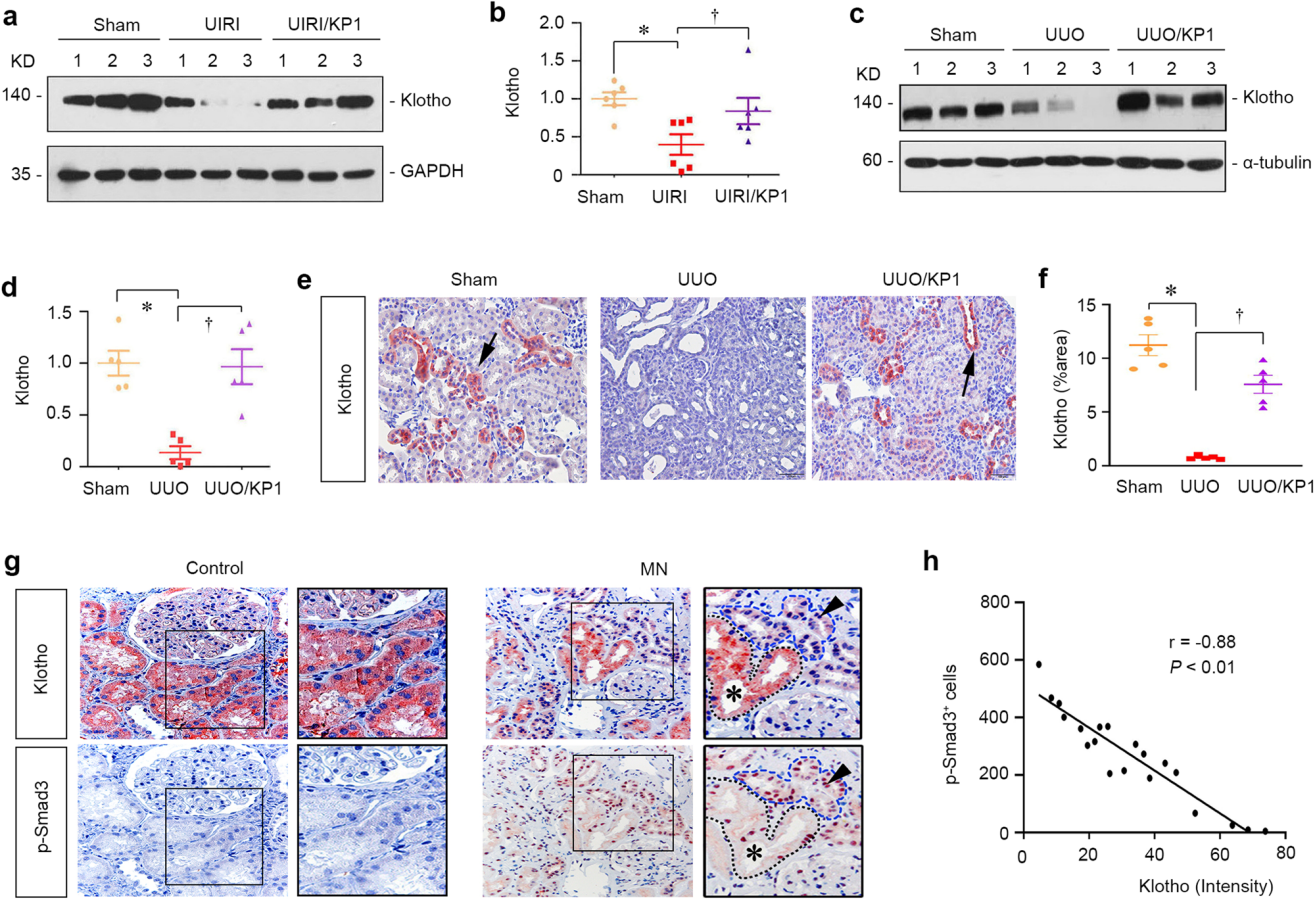
KP1 preserves the renal expression of endogenous Klotho after kidney injury. **a**, **b** KP1 restored endogenous Klotho expression after UIRI. Representative Western blot (**a**) and quantitative data of Klotho (**b**) are shown. *P* value (from left to right): 0.007, 0.037 (one-way ANOVA with Fisher’s LSD test). *n* = 6 biologically independent animals. Data are presented as mean values ± SEM. **c**, **d** KP1 preserved endogenous Klotho protein after UUO. Representative Western blot (**c**) and quantitative data of Klotho (**d**) are shown. *P* value (from left to right): <0.001, <0.001 (one-way ANOVA with Fisher’s LSD test). *n* = 5 biologically independent animals). Data are presented as mean values ± SEM. **e** Immunohistochemical staining for Klotho protein in the obstructed kidney at 7 days after UUO. Arrows indicate positive staining. Scale bar, 50 µm. **f** Quantitative data of Klotho positive staining area in each high-power field. *P* value (from left to right): 0.001, 0.003. *n* = 5 biologically independent animals. Data are presented as mean values ± SEM. **g**, **h** Colocalization of Klotho and p-Smad3. Representative micrographs show Klotho localized in those tubules with nuclei negative for expression of p-Smad3. Nontumor kidney tissue from the patients who had renal cell carcinoma and underwent nephrectomy was used as normal controls (**g**). Sequential paraffin-embedded kidney sections from patients with membranous nephropathy (MN) were immunostained for Klotho and p-Smad3. Scale bar, 50 μm. Boxed areas are enlarged. **h** Scatter plots with linear regression show an inverse correlation between Klotho expression levels and p-Smad3 positive cells in human kidney sections. The Spearman correlation coefficient (*r*) and two side *P* value are shown. Source data are provided as a Source Data file.

The kidney is the main source of endogenous Klotho in vivo. We wondered whether KP1 affects the expression of endogenous Klotho protein. To test this, we assessed the expression of Klotho protein in diseased kidneys in the absence or presence of KP1. As shown in Fig. 8a, b, Klotho was highly expressed and readily detectable by Western blotting in normal kidneys, and UIRI triggered the loss of renal Klotho expression. However, KP1 largely preserved renal expression of Klotho protein (Fig. 8a, b). Consistently, KP1 also preserved Klotho expression in the obstructed kidney after UUO (Fig. 8c, d). Immunohistochemical staining showed that Klotho was predominantly expressed in renal tubular epithelia in normal kidneys but completely lost in the obstructed kidney at 7 days after UUO, which was restored by KP1 (Fig. 8e, f). These data suggest that KP1 may elicit its protective activity at least partially through restoring endogenous Klotho expression.

To establish the clinical relevance of Klotho to TGF-β signaling in human CKD, we performed immunostaining for Klotho and p-Smad3 on serial sections of kidney specimens. As shown in Fig. 8g, abundant Klotho expression was evident in the tubular epithelium of normal human kidneys, whereas no or little p-Smad3^+^ cells were observed. However, Klotho expression was inhibited and Smad3 signaling was activated in human kidney biopsies from CKD patients with membranous nephropathy (MN). In tubules with high Klotho expression, a few p-Smad3^+^ cells were observed (Fig. 8g, asterisk), while in the adjacent tubules with low levels of Klotho, p-Smad3^+^ nuclei were abundant (Fig. 8g, arrowhead). There was clearly an inverse correlation between Klotho level and Smad3 activation in diseased kidneys (Fig. 8h), suggesting an intrinsic association of Klotho with TGF-β signaling in humans as well.

## Discussion

In this study, we report the identification of a Klotho-derived peptide that inhibits renal fibroblast activation in vitro and in vivo by targeting TGF-β1 signaling. We show that KP1 binds to TβR2 and competitively inhibits TGF-β1 engagement with TβR2. As such, KP1 effectively blocks Smad phosphorylation and nuclear translocation and represses the activation of several MAPK including ERK1/2, JNK, and p38. Delivery of KP1 by intravenous injections inhibits TGF-β signaling in vivo, mitigates fibrotic lesions after ischemic or obstructive injury, and restores endogenous Klotho expression. As KP1 is derived from Klotho, a naturally occurring anti-aging protein which expression is downregulated in all CKD tested^18,35^, it is conceivable to speculate that KP1 may elicit few, if any, adverse effects in vivo. The findings presented in the current study not only shed light on the mechanism by which Klotho ameliorates kidney fibrosis, but also provide an effective arsenal in the fight against fibrotic CKD.

The most interesting finding of the present study is the identification of KP1, a peptide with 30 amino acids, as the binding site to TβR2. Although Klotho is known to interact with TβR2 and inhibit TGF-β signaling^24^, the precise structural motif responsible for such binding was unknown. By screening a series of Klotho-derived peptides, we uncover that KP1, corresponding to the N-terminal region of Klotho ranging from Phe57 to Lys86, mediates the interaction between Klotho and TβR2. This conclusion is substantiated by several lines of evidence. First, KP1 by itself is sufficient and capable of binding to TβR2, as shown by co-immunoprecipitation, SPR analyses, and a microplate-based direct binding assay. Second, KP1 disrupts TGF-β and TβR2 engagement both in vitro and in vivo. Finally, KP1 competes with Klotho for binding to TβR2 (Fig. 2). Collectively, these findings highlight that KP1 harbors the binding domain of Klotho to TβR2. Our studies are also consistent with the notion that different regions of Klotho may elicit distinct actions.

As a large transmembrane protein, it is often considered to be challenging and expensive to produce and utilize Klotho as a therapeutic remedy. In addition, high serum Klotho levels are associated with hypophosphatemia and hypocalcemia due to the dysregulated calcium-phosphorus metabolism^36^. In this context, a small Klotho peptide, which retains the anti-fibrotic potential but eliminates undesirable effects, may offer distinguished advantages such as high potency and efficacy, reliable safety, well tolerability, and shorter time to market^37^. Indeed, the KP1 identified in the present study blocks fibroblast activation in vitro and ameliorates renal fibrosis in two well-characterized models induced by UIRI and UUO by intercepting TGF-β signal transduction (Figs. 4–7). As TGF-β is a key mediator in renal fibrosis by activating myofibroblasts and promoting excessive ECM deposition^7,9,10^, targeting TGF-β signaling has become a fundamental strategy for inhibiting tissue fibrosis in many organs^14,38,39^. It should be stressed that the anti-TGF-β effect of KP1 is robust and potent, as it inhibits TGF-β action in 5 different types of cells including rat kidney fibroblast cell line (NRK-49F), human proximal tubular epithelial cell line (HKC-8), mouse primary kidney tubular cells, rat primary cardiomyocytes, and cardiac fibroblasts, and it is more potent than ITD-1 both in vitro and in vivo. In addition, among diverse strategies for blocking TGF-β signaling, KP1 is quite unique in that it is derived from Klotho, a natural protein with anti-aging potential^40^. In this regard, our studies have suggested an effective and safe remedy for treating fibrotic CKD.

Another interesting finding in this study is that KP1 is preferentially targeted to and specifically accumulated in the injured kidney after intravenous injection, but not in the contralateral normal kidney and other major organs such as liver, lung, heart, and spleen (Figs. 4 and 6). Although a transient and low-level distribution of the FITC-labeled KP1 is initially observable in the lung as well, KP1 distribution is mainly restricted to the injured kidney within 0.5 h after tail vein injection, indicating the injured kidney as the primary organ for KP1 to target. The reason behind this specificity of KP1 distribution after intravenous injection is unknown. One potential explanation is due to the specific upregulation of TβR2, the binding partner of KP1, in the injured kidney (Figs. 5 and 7)^41–43^. Once KP1 binds to TβR2, it will be retained in the injured kidney leading to its local accumulation in situ (Fig. 6). Regardless of the mechanism, the very fact that KP1 is preferentially accumulated after systematic delivery highlights a targeted action of KP1 in diseased kidneys. Clearly, such a specific distribution of KP1 in the injured kidney is much desirable and advantageous, leading to selectively targeting hyperactive TGF-β signaling in vivo^44^.

The mechanism of KP1 action is clearly related to its binding and inhibition of TβR2, leading to the interception of TGF-β signaling. This notion is confirmed by the observations that KP1 blocks Smad and MAPK activation by TGF-β1 in vitro and by UIRI or UUO in vivo. However, it is conceivable that the action of KP1 in vivo may go beyond the simple inhibition of TGF-β/TβR2 engagement. In this regard, KP1 appears to inhibit TβR2 expression in vivo (Figs. 5 and 7), consistent with earlier studies showing that Klotho represses TβR2 expression^45^. This suggests that KP1 may inhibit TGF-β signaling not only through competing with TGF-β1 for binding to TβR2 but also by decreasing the expression of TβR2. More interestingly, when KP1 is given at 4 days after IRI at which time point Klotho is largely lost in this model^22^, it restores endogenous Klotho expression in vivo (Fig. 8). Although we cannot exclude the possibility that the restoration of endogenous Klotho expression by KP1 is due to the amelioration of kidney injury, KP1 may exert its protective action by specifically inducing Klotho expression, because KP1 does not affect the expression of total Smad2/3, ERK1/2, and p38 in diseased kidneys. Along this line, earlier studies show that TGF-β1 decreases the expression of Klotho by inducing H3K9 hypermethylation of *KL* gene^46,47^. Therefore, inhibition of TGF-β signaling by KP1 could restore Klotho expression by blocking H3K9 hypermethylation. Intriguingly, there is a close inverse relationship between Klotho level and Smad3 activation in human CKD (Fig. 8), providing the proof-of-principle that KP1 may mimic endogenous Klotho in antagonizing TGF-β signaling. More studies using large and diverse CKD populations are needed in this area.

The present study has its limitations and leaves questions unanswered. One unsolved issue is the details of KP1 and TβR2 interaction, which requires further sophisticated structural biology and site-directed mutation approaches. Notably, a large portion of KP1 peptide corresponds to one of the buried β-strands in the N-terminal domain of Klotho structure, suggesting that KP1 may only represent a portion of Klotho’s binding region for TβR2 and another region of Klotho could also be necessary for effective binding. Moreover, the quantitative values of the SPR data presented need to be independently confirmed. Future studies are warranted to decipher the molecular mechanism underlying KP1 and TβR2 interaction.

In summary, we report the discovery of a Klotho-derived peptide (KP1) that mimics the anti-fibrotic action of Klotho by constraining TGF-β signaling. Mechanistically, KP1 binds to TβR2, disrupts TGF-β and TβR2 engagement, and blocks Smad and MAPK signaling. Intravenous injection of KP1 results in its specific accumulation in the injured kidneys and ameliorates renal fibrotic lesions in different models of kidney fibrosis. As KP1 only contains 30 amino acids and targets specific to the diseased kidney, this peptide may provide a remedy for therapeutic intervention of fibrotic CKD. Although many studies are needed, it is hopeful that KP1 can be eventually translated into effective therapeutics for millions of CKD patients.

## Methods

### Peptide screening

The KL1 domain of Klotho was divided into 18 overlapping peptides encompassing about 30 amino acids each. Peptides were synthesized by GenScript (Piscataway, NJ) at >95% purity. The peptides were dissolved in 0.01 M acetic acid at 10 μg/μl concentration. NRK-49F cells were serum-starved for 24 h and then pre-incubated with each peptide at the concentration of (10 μg/ml) or otherwise indicated for 1 h before TGF-β1 (2 ng/ml) stimulation. Cells were collected at 24 h after TGF-β1 treatment. Whole-cell lysates were prepared and subjected to Western blot analyses.

### Animal models

Male *BALB/c* mice weighing 20–22 g were purchased from Beijing Vital River Laboratory Animal Technology Co. and housed in a standard environment which was characterized by 12 h light/dark cycle, 22–25 °C, and 40–60% humidity with free access to water and chow (catalog number: SWS9102). Mice have randomly divided into 3 groups: (i) sham controls; (ii) UIRI or UUO mice treated with vehicle; (iii) UIRI or UUO mice treated with KP1. A renal UIRI model was performed. Briefly, under general anesthesia, mice were made a midline abdominal incision, and the left kidney was clipped for 35 min by a vascular clamp. Mice were kept on the heating block at 37 °C during ischemia. The contralateral right kidney was removed on day 10 after ischemia–reperfusion. Mice were sacrificed 11 days after IRI. Blood and kidneys were collected for analysis. UUO model was established. Briefly, the left ureter of the lower pole of the kidney was obstructed by 3–0 surgical suture. KP1 peptide was dissolved in 0.01 M acetic acid and administrated intravenously via tail vein at concentration of 1 mg/day/kg (0.31 μM/day/kg) body weight for 6 days. For some experiments, ITD-1 (S6713, Selleck) was dissolved in PBS with 0.025% DMSO and 0.1% Tween 80 buffer and injected via tail vein at 0.31 μM/day/kg body weight. All animal studies were approved by the Laboratory Animal Ethics Committee of Nanfang Hospital, Southern Medical University.

### Cell culture and treatment

Normal rat kidney interstitial fibroblasts (NRK-49F) and human proximal tubular epithelial cells (HKC-8) were cultured at 37 °C. NRK-49F cells were serum-starved for 24 h and were pretreated with KP1 (10 µg/ml, 3 µM) for 1 h prior to incubation with TGF-β1 (#240-B; R&D Systems) at 2 ng/ml. Cells were collected at 45 min or 24 h after TGF-β1 treatment, respectively. Whole-cell lysates were prepared and subjected to Western blot analyses. For some experiments, cells were transiently transfected with expression vector human secreted Klotho (sKlotho) that only contains KL1 domain^48^, by using Lipofectamine 2000 reagent (Invitrogen, Grand Island, NY) prior to treatment with TGF-β1.

### Culture of primary cells

Mouse primary kidney tubular epithelial cells were isolated and cultured as previously described^49^. Briefly, the cortical part of mouse kidneys was minced, then digested with collagenase 4 for 40 min at 37 °C. The tubular tissues were centrifuged using 31% Percoll gradients for 10 min, resuspended, and washed twice with DMEM/F-12. Tubules were finally suspended in DMEM/F-12 supplemented with 10% bovine calf serum, 50 U/ml penicillin, and 50 mg/ml streptomycin. Cells were cultivated for 4–8 days. Tubular epithelial cells were characterized by morphology, positive staining for E-cadherin, and negative staining for vimentin, as shown in Supplementary Fig. 7.

Primary neonatal rat ventricular cardiomyocytes were isolated by enzymes digestion including tryptase and collagenase II, as reported elsewhere^50^. These primary cardiomyocytes exhibited spontaneous beating on the dish. The purity of the primary cardiomyocyte population was more than 95% based on the morphology and the staining for α-actin.

Primary cardiac fibroblasts were isolated from the hearts of neonatal rats^50^. Briefly, hearts were incised from rats in 3-day age, pre-digested in 4 °C with 0.25% trypsin for 12 h, followed by incubation with 1 mg/ml collagenase II to make cell suspension. Isolated cells were seeded in a culture plate and incubated for 90 min at 37 °C in a CO_2_ incubator. Adherent cells were cultivated in DMEM/F12 medium with 10% fetal bovine serum. Cardiac fibroblasts within three passages were subjected to various treatments as indicated. Primary cardiomyocytes and cardiac fibroblasts were treated with TGF-β1 (2 ng/ml) in the absence or presence of KP1 (3 µM). At different time points after treatment, cells were harvested for protein isolation and subjected to various analyses.

### qRT-PCR

Total RNA was isolated from NRK-49F cells by using a TRIzol RNA isolation system (Invitrogen, Carlsbad, CA). The cDNA was synthesized using 2 µg of RNA in 20 µl of reaction buffer containing AMV-RT (Promega, Madison, WI), random primers, and oligo primers at 42 °C for 60 min. Quantitative, real-time PCR (qRT-PCR) was performed using a Platinum SYBR Green qPCR Super Mix-UDG kit (Invitrogen). The sequences of the primer pairs: F: GAGGCACCACTGAACCCTAA; R: CATCTCCAGAGTCCAGCACA. QPCR data were collected by Step one software v2.2.2.

### Western blot analysis

Western blot analysis was performed as previously described^51^. The primary antibodies used were as follows: anti-fibronectin (dilution 1:10000; F3648; Sigma), anti-α-SMA (dilution 1:1000; ab7817; Abcam), anti-collagen-1 (dilution 1:1000; BM4017; Boster), anti-p-Smad3 (dilution 1:1000; #9520), anti-p-Smad2 (dilution 1:1000; #3104s), anti-p-ERK (dilution 1:1000; #9101s), anti-p-p38 (dilution 1:1000; #9211s), anti-p-JNK (dilution 1:1000; #9251s), anti-Smad2/3 (dilution 1:1000; #8685s), anti-JNK (dilution 1:1000; #9252), anti-ERK (dilution 1:1000; #4695), anti-p38 (dilution 1:1000; #8690; Cell Signaling Technology), anti-Klotho (dilution 1:1000; AF1819; R&D System), anti-TβR2 (dilution 1:1000; sc-7791; Santa Cruz), anti-TGF-β1 (dilution 1:1000; ab92486; Abcam), anti-GAPDH (dilution 1:5000; LK9002L; Sungene, Tianjin), anti-FITC (dilution 1:1000; PA1-26793; Invitrogen), anti-F4/80 (dilution 1:1000; 14-4801-82, eBioscience). The integration of all blots images was performed on Adobe Photoshop CS6. Graphpad Prism 8 was used to get statistics figures. Image J (v1.8.0) was used to quantify Western blot results.

### Co-immunoprecipitation

Co-immunoprecipitation (Co-IP) was performed as previously described^18^. Briefly, NRK-49F cell lysates were prepared and incubated overnight at 4 °C with FITC-KP1 (10 µg), anti-TβR2 antibody (Santa Cruz), and protein A/G plus agarose (sc-2003; Santa Cruz). The immunocomplexes were blotted with antibodies against FITC and TβR2, respectively. To determine whether KP1 or KP12 can disrupt the interaction between TGF-β1 and TβR2, cells were treated with TGF-β1 in the absence or presence of different amounts of KP1 or KP12 as indicated, and TGF-β1/TβR2 interaction was then assessed. Similarly, to determine whether KP1 can disrupt the interaction between Klotho and TβR2, cells were transfected with sKlotho plasmid for 24 h and then incubated with TGF-β1 in the absence or presence of KP1, followed by analyzing the interaction between sKlotho and TβR2.

### Affinity measurements by Biacore T200

The SPR analyses were performed on a Biacore T200 apparatus (GE Healthcare, Uppsala Sweden) at 25 °C. Recombinant TGF-β receptor 2 protein (43.4 kDa, >95% as determined by SDS-PAGE) (10358-H03H-B; Sino Biological Inc., Beijing, China) diluted at 50 µg/ml in 10 mM acetate pH 4.0, was covalently immobilized on a CM5 sensor chip (GE Healthcare) using amine-coupling chemistry. Samples of KP1 (3.229 kDa, HPLC purity >95%) or KP12 (3.6 kDa, HPLC purity >95%) in PBS at various concentrations were injected at a flow rate of 60 μl/min. The injection time was set to 180 s, the dissociation time was set to 540 s, and 50 mM Gly-HCl (pH = 2.5) was used as the regeneration buffer. Data were fitted to a 1:1 binding model to obtain K_D_. All results presented were representative of double or triplicated injections in an interleaved manner. Two independent experiments were performed.

### KP1 and TβR2 direct binding assay

We developed a simple microplate-based, fluorescence assay to detect KP1 and TβR2 direct binding in vitro. Human TβR2 (10358-H08B; Sino Biological Inc.) was diluted in coating buffer (C3041-100CAP; Sigma) at 10 μg/ml and coated on 96-well microplate (100 μl/well) at room temperature for 1.5 h, followed by washing with 200 μl of washing buffer for four times. After blocking with 200 μl of blocking buffer (2% BSA in washing buffer) for 1 h, FITC-KP1 or FITC-KP12 was diluted at a concentration of 50 μg/ml and added to each well (100 μl/well). After incubating at room temperature for 1 h, the microplates were washed four times, and fluorescence intensity was read by using BioTek Synergy HTX Multimode Microplate reader at an excitation wavelength of 485/20 nm and emission wavelength of 528/20 nm. All procedures from the addition of the FITC-conjugated peptides were carried out in the dark. The relative fluorescence intensity (arbitrary units) was reported. As a negative control, BSA-coated microplates were used.

### Organ imaging of KP1 distribution

*BALB/c* mice were subjected to UUO and UIRI, respectively. Seven days after surgery, mice were intravenously injected with 400 µl of FITC labeled KP1 (5 mg/kg) or vehicle. All mice were sacrificed after 30 min. Major organs including kidneys, heart, liver, lung, and spleen were removed and placed in glass dishes. Organs were exposed to a Bruker FX PRO imaging system equipped with an excitation at 470 nm and emission at 535 nm, and images were taken with a camera and digitally analyzed. All procedures were conducted in dark.

### Liquid chromatography–tandem mass spectrometry

The left kidneys of *BALB/c* mice were subjected to UUO surgery. Organs were obtained 0.5 h after intravenous injection with KP1 (5 mg/kg body weight). Tissues were weighed and lysed with PBS (10 µl per milligram weight) on ice. The supernatants were collected after centrifugation at 10,000 *g* at 4 °C for 10 min. KP1 levels in organs were quantified by LC–MS/MS assay. The LC–MS/MS system consisted of Agilent 6460 Triple Quadrupole LC–MS/MS systems via an ESI interface. A volume of 50 ul of tissue homogenate was combined with 100 μl of acetonitrile containing 1 μg/ml of internal standard and then centrifuged at 14,000 *g* for 15 min. After an additional centrifugation step, the supernatant was analyzed after injection into an Agilent Poroshell 120 EC-C18 column (3.0 × 50 mm, 2.7 μm).

### Histology and immunostaining

Paraffin kidney sections were prepared by a routine procedure. Kidney sections (2 µm thickness) were subjected to MTS for assessing collagen deposition and fibrotic lesions. Quantification of the fibrotic lesion was carried out by image J (v1.8.0) software, and at least three randomly chosen images were analyzed per mouse. Immunohistochemical staining was performed with 3 µm kidney sections according to the established protocol^18^. The antibodies against fibronectin (dilution 1:50; F3648), α-SMA (dilution 1:50; ab7817), p-Smad3 (dilution 1:50; #9520), anti-CD3 (dilution 1:50; ab16669; Abcam), anti-F4/80 (dilution 1:50; MCA497; BIO-RAD), anti-vimentin (dilution 1:50; #5741s; Cell Signaling Technology), anti-Klotho (dilution 1:50; AF1819; R&D System) and p-ERK1/2 (dilution 1:50; #9101s) were used. Some samples were subjected to immunofluorescence staining, according to procedures described previously^18^. Briefly, NRK-49F cells were cultured on coverslips and fixed with cold methanol: acetone (1:1) for 15 min. The slides were immunostained with primary antibodies against fibronectin (dilution 1:50; F3648) and p-Smad3 (dilution 1:50; #9520) overnight and then stained with donkey anti-rabbit IgG (H + L) (711-165-152, Jackson ImmunoResearch Laboratories). For the FITC-KP1 distribution assay, tissue cryosections were fixed with 3.7% paraformalin for 15 min at room temperature. The slides were immunostained with primary antibodies against FITC (PA1-26793; Invitrogen) and then stained with a Cy3-conjugated secondary antibody.

Human kidney specimens were obtained from diagnostic renal biopsies performed at the Nanfang Hospital, Southern Medical University, with written informed consent from the patients. Non-tumor kidney tissue from the patients who had renal cell carcinoma and underwent nephrectomy was used as normal controls. Kidney biopsies from patients with MN and IgA nephropathy were used. These patients are males aged 34–58. Paraffin-embedded human kidney biopsy sections were used for immunohistochemical staining. All the studies involving human samples were approved by the Medical Ethics Committee of the Nanfang Hospital, Southern Medical University.

### Statistics and reproducibility

All data were expressed as mean with SEM. Statistical analysis of the data was carried out using IBM SPSS Statistics 22. The detailed statistical test used for each experiment were stated in figure legends. *P* < 0.05 was considered statistically significant. The representative figures shown in paper were repeated at least three independent experiments.

### Reporting summary

Further information on research design is available in the Nature Research Reporting Summary linked to this article.

## Supplementary information

Supplementary Information

Reporting Summary

## Data Availability

Source data are provided with this paper. All data generated in this study are provided in the Supplementary Information/Source Data file. Source data are provided with this paper.

## Source data

Source Data
