# Author Correction: A Klotho-derived peptide protects against kidney fibrosis by targeting TGF-β signaling

**DOI:** 10.1038/s41467-022-34454-8

**Published:** 2022-11-04

**Authors:** Qian Yuan, Qian Ren, Li Li, Huishi Tan, Meizhi Lu, Yuan Tian, Lu Huang, Boxin Zhao, Haiyan Fu, Fan Fan Hou, Lili Zhou, Youhua Liu

**Affiliations:** 1 State Key Laboratory of Organ Failure Research, National Clinical Research Center of Kidney Disease, Division of Nephrology, Nanfang Hospital, Southern Medical University, Guangzhou, China; 2 Analysis and Test Center, Guangdong University of Technology, Guangzhou, China; 3 Department of Pharmacy, Nanfang Hospital, Southern Medical University, Guangzhou, China; 4 Bioland Laboratory (Guangzhou Regenerative Medicine and Health Guangdong Laboratory), Guangzhou, China; 5 Department of Pathology, University of Pittsburgh School of Medicine, Pittsburgh, PA USA

**Keywords:** Peptide delivery, Drug discovery, Kidney diseases

Correction to: *Nature Communications* 10.1038/s41467-022-28096-z, published online 21 January 2022

The original version of this article contained an error in the Results section, where it stated: ‘After several rounds of screening, we identified a peptide with 30 amino acids encompassing from Phe57 to Lys86 of Klotho protein (Fig. 1a)’. This should read: ‘After several rounds of screening, we identified a peptide with 30 amino acids encompassing from Phe57 to Gly86 of Klotho protein (Fig. 1a)’. This has been corrected in both the PDF and HTML versions of the Article.

