# Supplementary material for: A Klotho-derived peptide protects against kidney fibrosis by targeting TGF-β signaling
Source: Nat Commun. 2022 Jan 21;13:438. doi: 10.1038/s41467-022-28096-z (PMC8782923; doi:10.1038/s41467-022-28096-z)
Supplement: Supplementary file 1 — Supplementary Information [file 41467_2022_28096_MOESM1_ESM.pdf]

A Klotho-derived peptide protects against kidney fibrosis by targeting TGF-β signaling

Qian Yuan[1], Qian Ren[1], Li Li[1], Huishi Tan[1], Meizhi Lu[1], Yuan Tian[1], Lu Huang[2], Boxin Zhao[3], Haiyan Fu[1], Fan Fan Hou[1,4], Lili Zhou[1,4*], Youhua Liu[1,4,5*]

Supplementary data

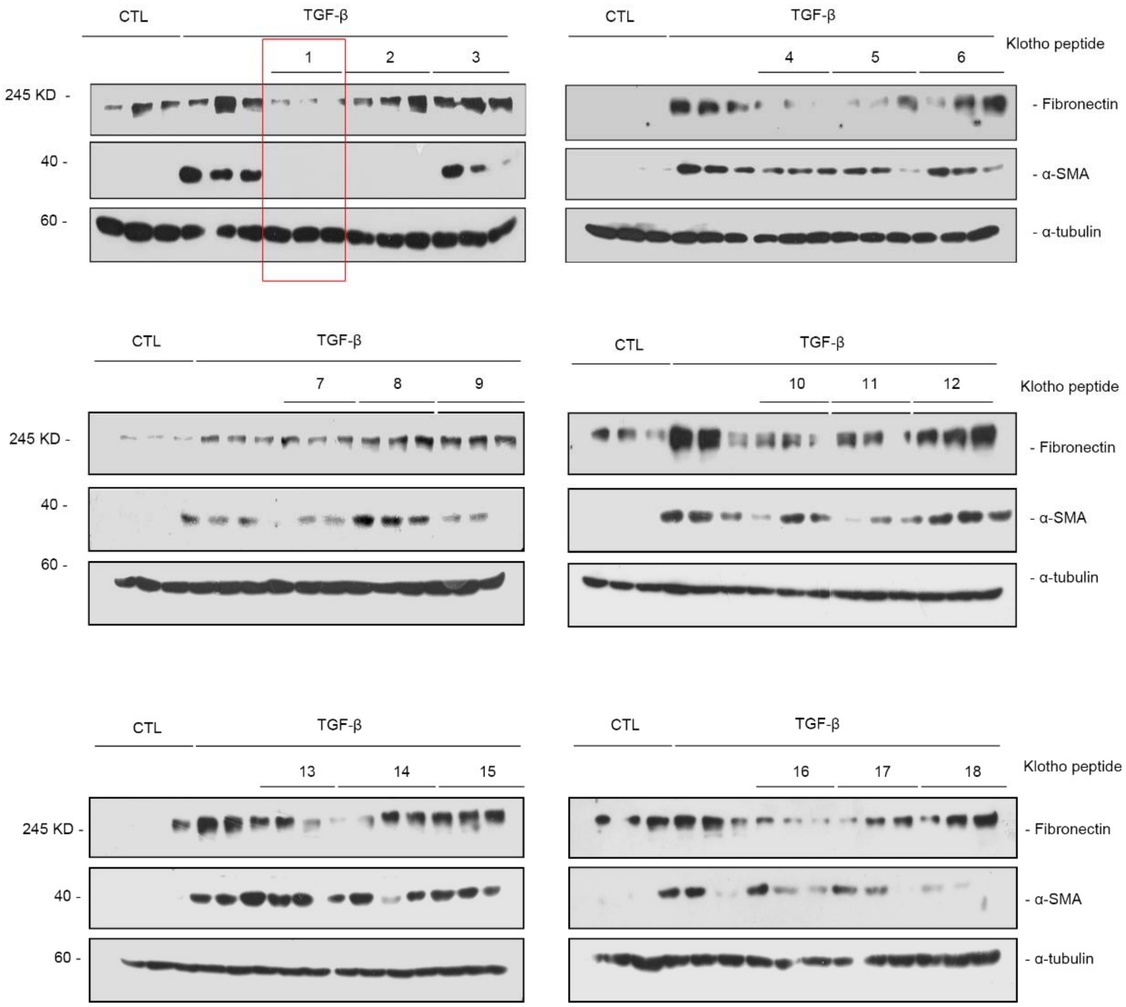

Supplementary Fig. 1. Screening of Klotho-derived peptide mini-library. NRK-49F cells were treated with various peptides at the concentration of 10 ug/ml, followed by incubation with TGF-β1 (2 ng/ml) for 24 hours. Cell lysates were immunoblotted for fibronectin and α-SMA expression. KP1 was identified as the most potent peptide that inhibited TGF-β1-induced fibronectin and α-SMA expression

in NRK-49F cells. Numbers (1 to 18) indicate each individual peptide.

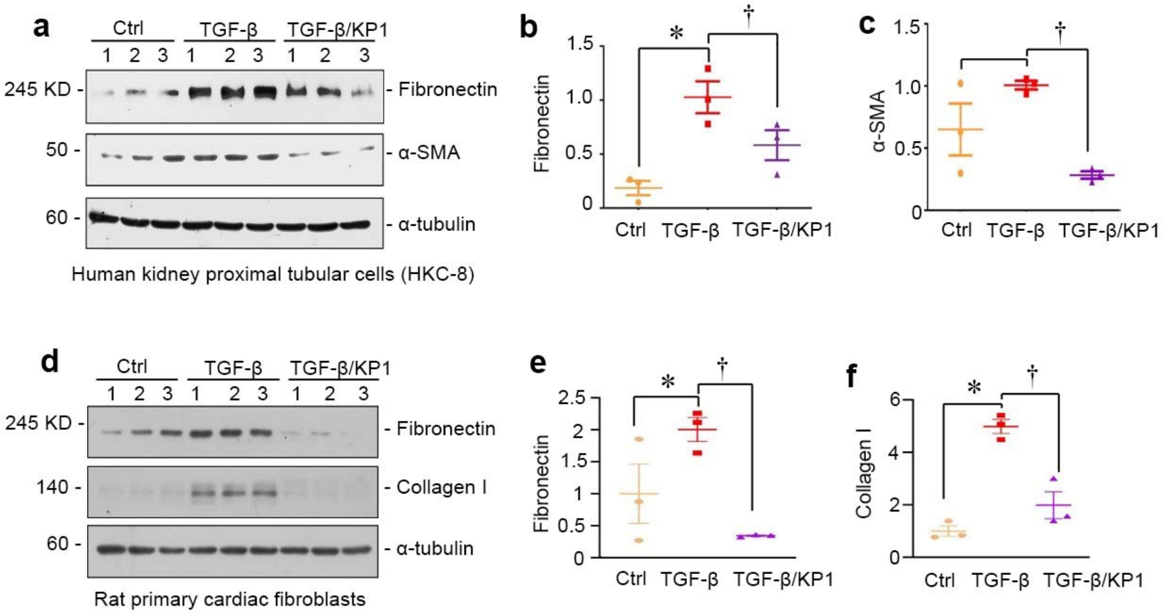

Supplementary Fig. 2. KP1 inhibits fibrotic responses induced by TGF-β1 in human kidney proximal tubular epithelial cells (HKC-8) and rat primary cardiac fibroblasts. (**a**) Representative Western blotting showed that KP1 inhibited TGF-β1-induced fibronectin and a-SMA expression in HKC-8 cells. (**b**, **c**) Quantitative data showed that KP1 inhibited TGF-β-induced fibronectin (**b**) and α-SMA (**c**) expressions. *P* values (from left to right): 0.003 and 0.044 (fibronectin); 0.087 and 0.006 (α-SMA). n=3 biologically independent cells. (**d-f**) Representative Western blot **(d)** and quantitative data of fibronectin **(e)** and collagen I **(f)** in primary cardiac fibroblasts are shown. *P* values (from left to right): 0.048 and 0.006 (fibronectin); <0.001 and 0.001 (collagen I). n=3 biologically independent cells. *P* values were calculated by one-way ANOVA followed by Fisher's Least-significant Difference (LSD) post hoc test. Data are presented as mean values +/- SEM.

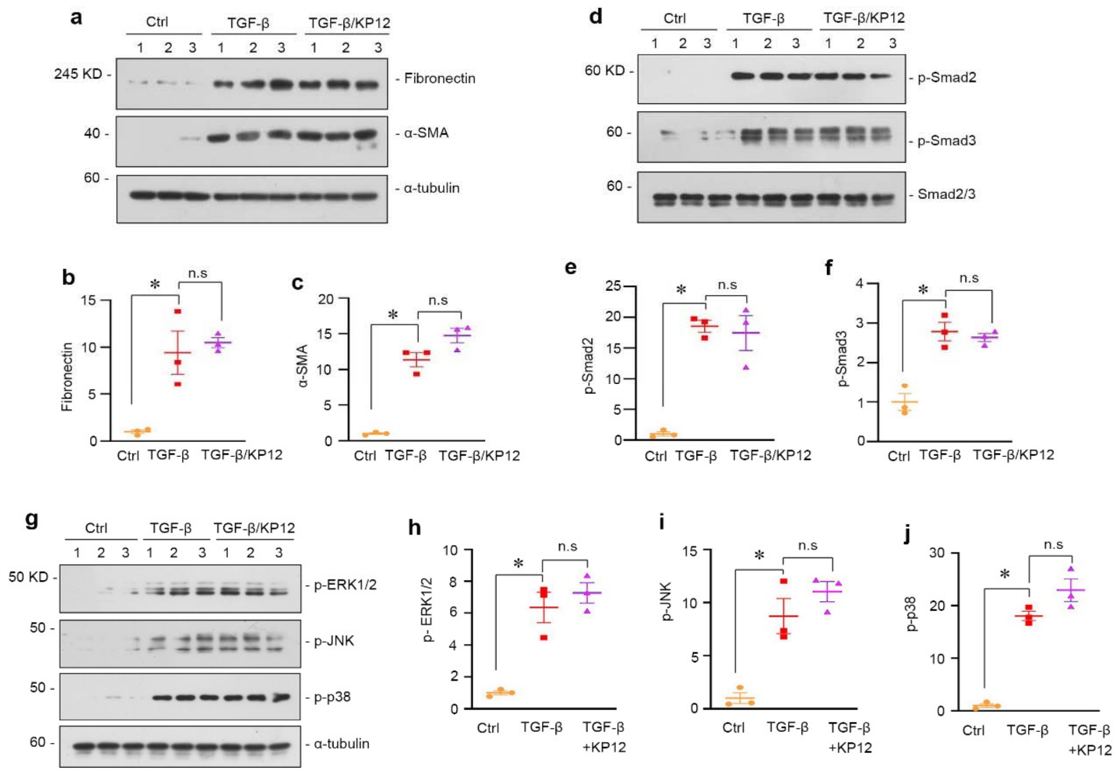

Supplementary Fig. 3. Negative peptide KP12 does not inhibit TGF-β signaling. **(a)** KP12 did not inhibit protein expression of fibronectin and α-SMA. NRK-49F cells were preincubated with KP12 (10 μg/ml) for 1 hour and then treated with TGF-β1 (2 ng/ml) for 24 hours. **(b, c)** Quantitative data of fibronectin **(b)** and α-SMA**(c)**. *P* values (from left to right): 0.005, 0.602 (fibronectin); 0.017, 0.176 (α-SMA). n=3 biologically independent cells. **(d)** KP12 did not inhibit the phosphorylation of Smad2 and Smad3 induced by TGF-β1. Serum-starved NRK-49F cells were pre-treated with KP12 or vehicle for 1 hour and then stimulated by TGF-β1 for 45 min. **(e, f)** Quantitative data of p-Smad2 **(e)** and p-Smad3 **(f)** are presented. *P* values (from left to right): 0.003, 0.972 (p-Smad2); 0.001, 0.594 (p-Smad3). n=3 biologically independent cells. **(g-j)** KP12 did not inhibit TGF-β1-induced phosphorylation of ERK1/2, JNK and p38. Western blot analyses **(g)** and quantitative data of p-ERK1/2 **(h)**, p-JNK **(i)** and p-p38 **(j)** are shown. *P* values from left to right): 0.001, 0.372 (p-ERK1/2); 0.003, 0.202 (p-JNK); 0.002, 0.293 (p-p38). n=3 biologically independent cells. Ctrl, control. Data are presented as mean values +/- SEM. *P* values were determined by one-way ANOVA with Fisher's LSD post hoc test in (**b**, **f**, **h**, **i**) or one-way ANOVA with Dunnett's T3 test in (**c, e, j**).

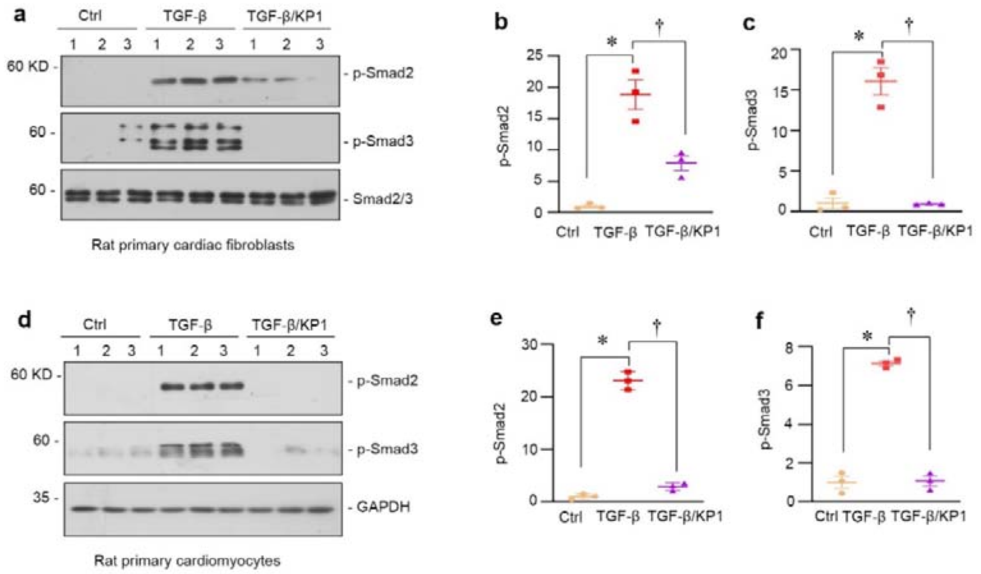

Supplementary Fig. 4. KP1 inhibits TGF-β signaling in primary cardiac fibroblasts and cardiac cells. Primary cardiac fibroblasts and primary cardiac cells were pre-incubated with KP1 (10 μg/ml) for 1 hour and then treated with TGF-β1 (2 ng/ml) for 45 minutes. **(a-c)** KP1 inhibited TGF-β1-induced phosphorylation of Smad2 and Smad3 in rat primary cardiac fibroblasts. Western blot analyses **(a)** and quantitative data of p-Smad2 **(b)** and p-Smad3 **(c)** are shown. *P* values (from left to right): <0.001, 0.002 (p-Smad2); 0.014, 0.025 (p-Smad3). n=3 biologically independent cells. **(d-f)** KP1 inhibited TGF-β1-induced phosphorylation of Smad2 and Smad3 in rat primary cardiac cells. Western blot analyses **(d)** and quantitative data of p-Smad2 **(e)** and p-Smad3 **(f)** are shown. *P* values (from left to right): <0.001, <0.001 (p-Smad2); <0.001, <0.001 (p-Smad3). n=3 biologically independent cells. Ctrl, controls. Data are presented as mean values +/- SEM. *P* values were determined by one-way ANOVA with Fisher's LSD post hoc test.

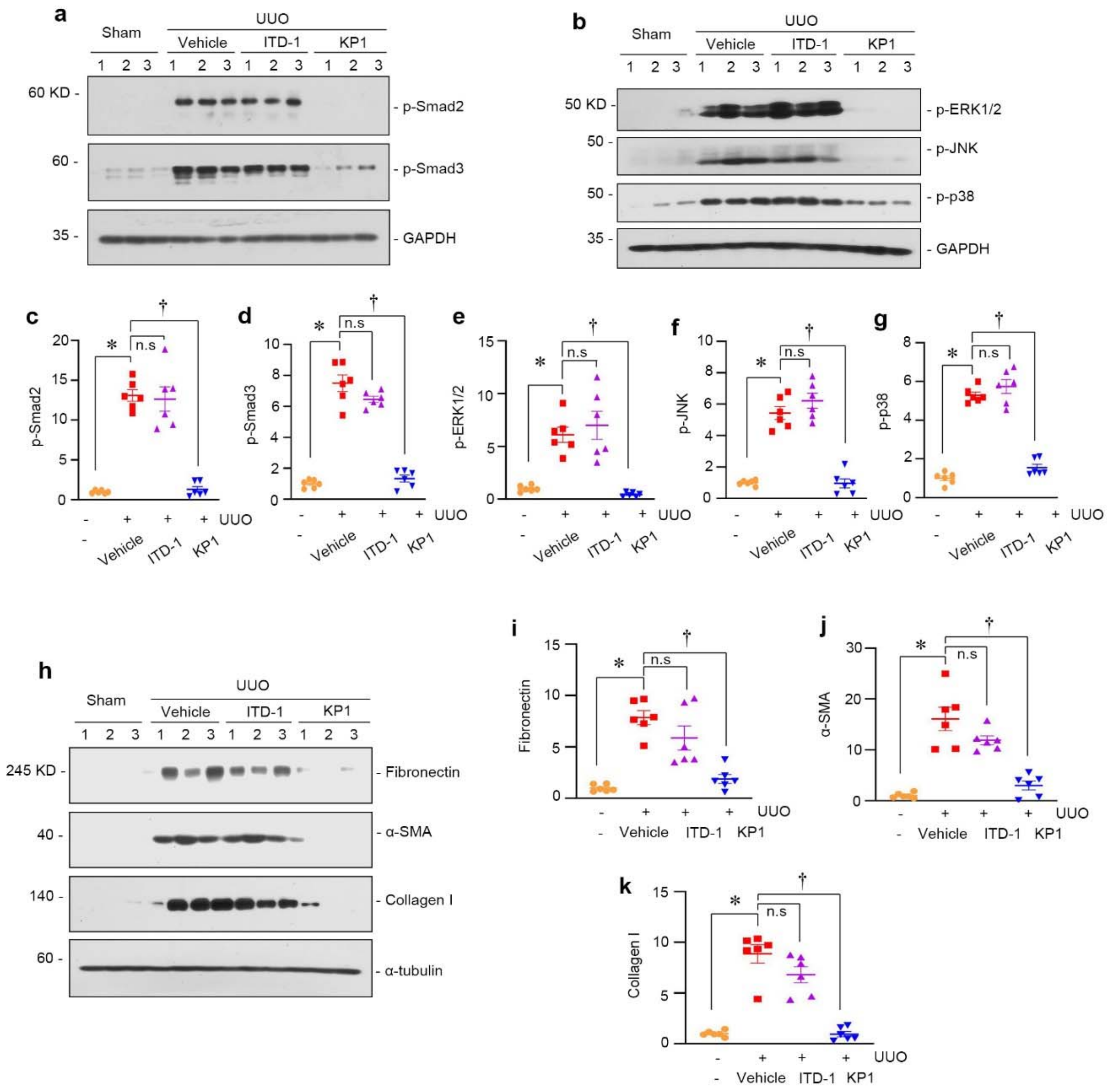

Supplementary Fig. 5. KP1 is more potent than ITD-1 in inhibiting TGF-β signaling and renal fibrosis *in vivo*. UUO mice were treated with KP1 (1 mg/kg, 0.31 μM/kg) or ITD-1 (0.31 μM/kg). Western blot analyses **(a, b)** and quantitative data of p-Smad2 **(c)**, p-Smad3 **(d)**, p-ERK1/2 **(e)**, p-JNK **(f)** and p-p38 **(g)** are shown. *P* values (from left to right): <0.001, 1.00, <0.001 (p-Smad2); <0.001, 0.438, <0.001 (p-Smad3); 0.004, 0.988, 0.002 (p-ERK1/2); <0.001, 0.758, <0.001 (p-JNK); <0.001, 1.00, 0.001 (p-p38). n=6 biologically independent animals. **(h-k)** Western blot analyses **(h)** and quantitative data of fibronectin **(i)**, α-SMA **(j)** and Collagen I **(k)** are shown. *P* values (from left to right): 0.001, 0.627, <0.001 (fibronectin); 0.006, 0.497, 0.007 (α-SMA); 0.001, 0.477, 0.001 (collagen I). n=6 biologically independent animals. Data are presented as mean values +/- SEM. Statistical significance was determined by one-way ANOVA followed by Dunnett's T3 post hoc test.

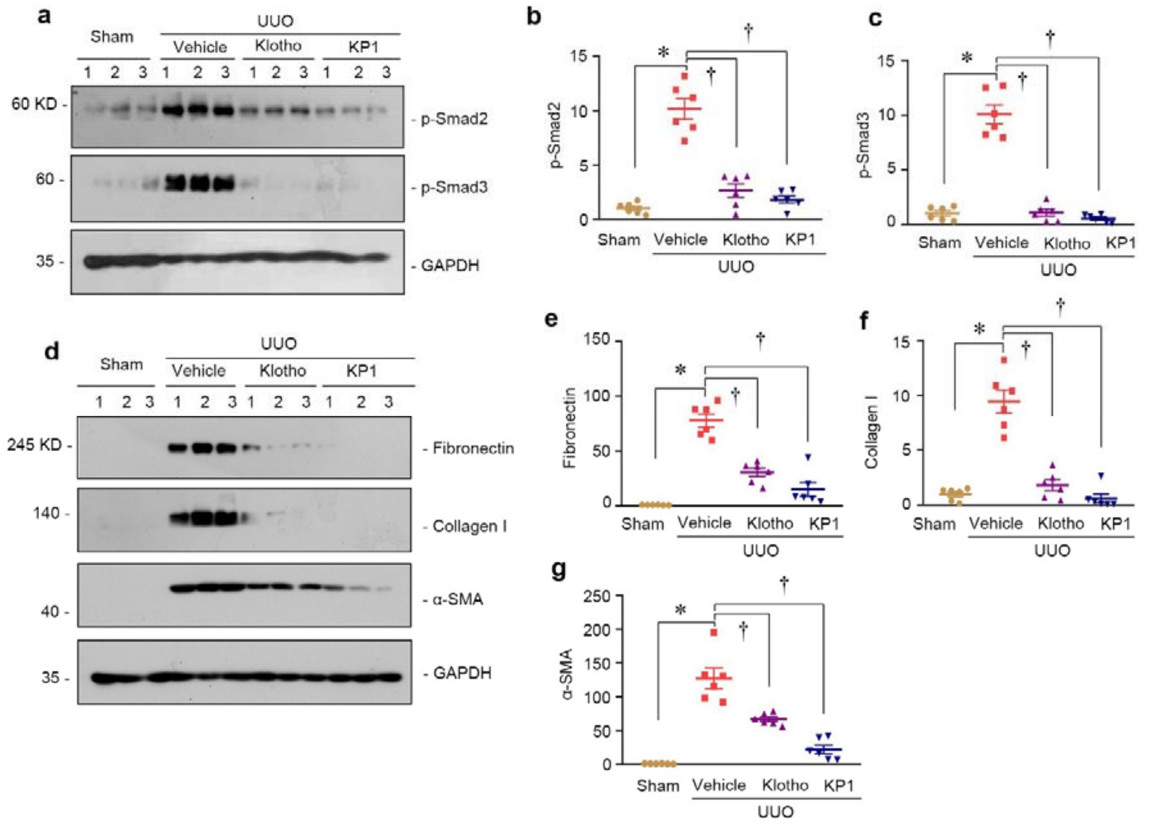

Supplementary Fig. 6. KP1 is comparable to Klotho in inhibiting TGF-β signaling and renal fibrosis *in vivo*. UUO mice were treated with KP1 (1 mg/kg) or soluble Klotho (10 µg/kg). Western blot analyses **(a)** and quantitative data of p-Smad2 **(b)**, p-Smad3 **(c)** are shown. *P* values (from left to right): 0.001, 0.001, 0.001 (p-Smad2); <0.001, <0.001, <0.001 (p-Smad3). n=6 biologically independent animals. **(d-g)** Western blot analyses **(d)** and quantitative data of fibronectin **(e)**, collagen I **(f)** and α-SMA **(g)** are shown. *P* values (from left to right): <0.001, <0.001, <0.001 (fibronectin); 0.002, 0.002, 0.001 (collagen I); 0.002, 0.044 ,0.002 (α-SMA). n=6 biologically independent animals. Data are presented as mean values +/- SEM. *P* values were calculated by one-way ANOVA with Dunnett's T3 post hoc test.

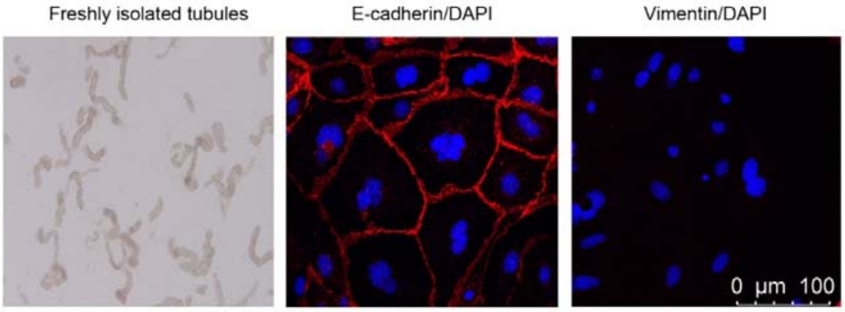

Supplementary Fig. 7. Characterization of mouse primary kidney tubular epithelial cells. Representative micrographs showed freshly isolated renal tubules, immunofluorescence staining of E-cadherin and vimentin. DAPI indicates nuclear staining.

Supplementary Table 1. Binding affinity analyzed by SPR

Analyte	k_a ($M^{-1}s^{-1}$)	k_d (s^{-1})	K_D (μM)
KP1	407.7	5.78* 10^{-4}	1.41
KP12	159.5	2.33* 10^{-3}	14.6

Supplementary Table 2. Blood phosphorus and calcium levels in different groups

Group	Phosphorus (mmol/L)	Calcium (mmol/L)
Sham	3.58 ± 0.4	2.1 ± 0.16
UIRI	5.13 ± 1.12 *	2.78 ± 0.37 *
UIRI+KP1	5.0 ± 1.06 *	2.59 ± 0.2 *

*$P <$ 0.05 versus Sham

P values of phosphorus: Sham vs UIRI, *P*<0.001; UIRI vs UIRI/KP1, *P*=0.220. *P* values of calcium: Sham vs UIRI, *P*=0.011; UIRI vs UIRI/KP1, *P*=0.798. (n=6 animals). *P* values were determined by one-way ANOVA with Fisher's LSD post hoc test. Data are presented as mean values +/- SEM.
